# PPAR Signaling Maintains Metabolic Homeostasis under Hypothermia in Freshwater Drum (*Aplodinotus grunniens*)

**DOI:** 10.3390/metabo13010102

**Published:** 2023-01-08

**Authors:** Ningyuan Wu, Haibo Wen, Pao Xu, Jianxiang Chen, Miaomiao Xue, Jianlin Li, Meiyao Wang, Changyou Song, Hongxia Li

**Affiliations:** 1 Freshwater Fisheries Research Center, Chinese Academy of Fishery Sciences, No. 9 Shanshui East Road, Wuxi 214081, China; 2 Wuxi Fisheries College, Nanjing Agricultural University, Wuxi 214081, China

**Keywords:** *Aplodinotus grunniens*, hypothermia, lipid metabolism, amino acid metabolism, PPAR signaling

## Abstract

*Aplodinotus grunniens*, known as freshwater drum, is a kind of eurythermal freshwater fish that is widely distributed in North America. In 2019, our research group reached a milestone on its artificial breeding and cultivation and have investigated its physiological adaption to the environment, providing a breakthrough and prospects for aquaculture. However, its adaptability and metabolic homeostasis to hypothermia is not fully understood. In this experiment, cold stress was conducted at 18 °C (LT18) and 10 °C (LT10) with 25 °C as control (Con) for 8 days to explore the effects of short-term hypothermia on the physiology and metabolism of freshwater drum. From the results, the level of free essential amino acids in LT18 and LT10 decreased significantly after 2 days cold stress compared with Con. Furthermore, plasma total triglyceride (TG) content and lipase (LPS) activity were decreased at LT10 for 2d. With RNA-seq in the liver, metabolic-related signaling, especially amino acid synthesis and lipid metabolism, was inhibited by hypothermia. Specifically, the PPAR signaling pathway is correlated with the inhibition of lipid and amino acid metabolism induced by hypothermia. These data confirmed that PPAR signaling maintains lipid and amino acid metabolic homeostasis during cold stress. These results give a theoretical foundation for hypothermia resistance in the area of metabolic homeostasis for freshwater drum.

## 1. Introduction

Fish are ectotherms, and temperature alternations in the living surroundings have many physiological effects on fish feeding, growth, development, immunity, and reproduction [1,2,3]. Different fish species have their own suitable living temperature. Extreme low or high temperature will directly or indirectly affect physiological function and biochemical metabolism [4,5]. Therein, cold stress is a kind of severe stress that could induce physiological dysfunction and even mortality for aquatic animals [6,7].

Studies on fish have suggested that temperature is interrelated to their digestion and metabolism of fish. Under low temperature, glycogen catabolism expedites in fish tissues to meet the increasing energy requirements of resistance to cold stress [8]. At the same time, cold stress leads to an increase in cortisol level, which is released into the blood, thereby inducing gluconeogenesis and significantly increasing the plasma glucose concentration [9]. However, as the cold stress continues, plasma glucose will gradually drop below normal levels [10]. Under low-temperature stimulation, the biological enzyme activity will also be affected, resulting in reduced cellular metabolism and protein synthesis [11]. Additionally, low-temperature stress can increase the activity of stearoyl-CoA desaturase (SCD) and Δ6 and Δ9 fatty acid desaturase, resulting in changes in fatty acid composition [12,13], and promoting the synthesis of unsaturated fatty acids in fish [14,15]. Moreover, it can also increase the level of oxidized lipids, which could induce the dysfunction of the cell [16].

Peroxisome proliferators-activated receptors (PPARs) are a class of nuclear receptor transcription factors which are ligand activated. PPARs play a significant role in adipogenesis, lipid metabolism, as well as maintenance of metabolic homeostasis, and are associated with a variety of metabolic related diseases, such as diabetes [17,18,19]. Additionally, it has been reported that PPARα also plays a role in in regulating the oxidation of fatty acids and amino acid metabolism [20,21].

*Aplodinotus grunniens* is widely distributed in North America, as far north as the Great Lakes region of Canada and as far south as Mexico and Guatemala [22]. Such latitude distribution indicates *A. grunniens* could adapt to a wide range of water temperatures. *A. grunniens* has a high edible proportion due to its thick back muscles, and the flesh is delicious, nutritious, abundant in protein, amino acids, and fatty acids [23,24,25]. These visible characteristics show the potential of *A. grunniens* cultivation to offer high-quality proteins to human beings [23,24,25]. Nevertheless, there are still limited resources in domestication, aquaculture, and management practices for the species. Furthermore, little scientific research has been carried out in this area. For these promising foregrounds, we introduced the *A. grunniens* larval form USA in 2016 and made a significant breakthrough on the artificial breeding and cultivation three years later. This offered a great progress and prospect for aquaculture [25,26,27]. It is reported that *A. grunniens* can reproduce under temperatures between 22 °C and 30 °C [28]. Early studies also showed that the minimum value of its survival temperature may be close to 1 °C [29] and its growth tends to be inhibited when exposed to living surroundings under 14 °C [30]. Additionally, our previous study reveals that hypothermia dysregulated glucose and lipid metabolism, and thereby induced antioxidant and immune dysfunction under 10 °C in freshwater drum. However, the underlying mechanism of hypothermia on metabolic homeostasis remains unclear. Based on our previous experiments and fish samples, 10 °C (LT10) was selected as the minimum temperature, and 18 °C (LT18) was selected as the intermediate temperature, while 25 °C was selected as the control temperature (Con) in this study. The impact of hypothermia on the physiological metabolism of freshwater drum were studied by comparing the differences of plasma biochemical parameters index, fatty acid and amino acid composition, as well as the high-throughput RNA-seq among different groups. Moreover, PPARs signaling undertaken in metabolic homeostasis was emphatically explored. Our study will provide a basis for the adaptation of freshwater drum to adverse temperatures in future production and breeding.

## 2. Materials and Methods

### 2.1. Ethics Statement

Our experiment was approved by the Animal Care and Use Committee of Nanjing Agricultural University (Nanjing, China). All animal procedures were carried out in terms of the Guideline for the Care and Use of Laboratory Animals in China.

### 2.2. Experimental Animals and Rearing Conditions

**Figure 1:**
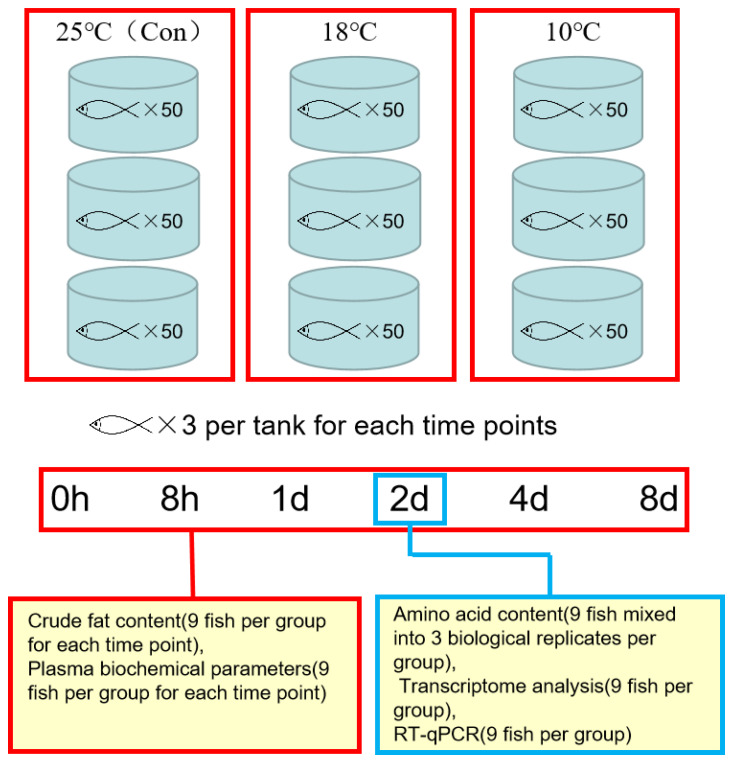
Overview of the experimental outline.

The experiment was conducted in Wuxi Fisheries College of Nanjing Agricultural University. The second-generation larvae were used as the laboratory fish. First-generation parents were introduced from the USA by Freshwater Fisheries Research Center, Chinese Academy of Fishery Sciences (Wuxi, China). The fish were raised in an indoor circulating water system, in which temperature could be adjusted according to experimental requirements (specifications for φ 820 × 700 mm). The initial body weights of the freshwater drums were 20.88 ± 2.75 g. They were stochastically assigned to nine tanks with a density of 50 fish per tank (Figure 1). The fish fed with fresh shrimp (3–5% body weight) daily were acclimated for 7 days under 25 °C prior to the experiment. After acclimation, the water temperature was set to 25 ± 1 °C, 18 ± 1 °C, and 10 ± 1 °C, and each treatment had three replicates. The normal water temperature was 25 ± 1 °C, while 18 °C and 10 °C were gradually achieved by the temperature-adjustable circulating water system within 6 h. Dissolved oxygen was >6 mg/L, pH was 7.2–7.8, and NH_3_ was <0.05 mg/L throughout the whole experiment.

### 2.3. Sample Collection

We conducted the experiments by collecting samples, respectively at 8 h, 1 day, 2 days, 4 days, and 8 days after cold stress. At each time point, nine fish (3 fish per tank) were selected to collect samples at random. We used MS-222 (0.1 g/L) to anaesthetize the fish (Figure 1). Before extracting the plasma (centrifugation at 5000 rpm for 10 min at 4 °C), blood samples were taken from caudal vein. For the purpose of composition and digestive enzyme activity index measurement, the plasma samples were stored at −80 °C. Similarly, the liver tissue was immediately frozen with liquid nitrogen and kept at −80 °C for the following analyses. The fish were dissected and sampled on ice.

### 2.4. Crude Fat Content Determination

Nine fish per group (3 fish per tank) for each time point were taken for body composition determination (Figure 1). Crude fat content (CFC) of each fish was determined by Soxhlet extraction method [31].

### 2.5. Plasma Biochemical Parameters Index Analysis

Plasma samples were used to measure TC (total cholesterol,), TG (total triglyceride), LPS (lipase activity), GLU(Glucose), AMS (α-amylase activity), TP (total protein), TPS (trypsin activity) following the instructions (Figure 1). In detail, TC were determined by COD-PAP method, TG by GPO-PAP method, LPS by colorimetric method, GLU by glucose-oxidase method, AMS by starch-iodine colorimetric method, TP by Coomassie brilliant blue method, TPS by UV-spectrocolorimetry method. Assay kits used in this study were all purchased from Nanjing Jiancheng Bioengineering Institute, China. The product numbers are as follows: TC, A111-1-1; LPS, A054-2-1; GLU, F006-1-1; AMS, C016-1-1; TP, A045-3-2; TPS, A080-2-2; TG, A110-1-1. The operations were strictly carried out according to the instructions.

### 2.6. Hydrolyzed and Free Amino Acid Content Analysis at 2 Days

Three fish were randomly selected out of the nine which were collected after 2 days’ cold stress (Figure 1), the muscles on both sides of the spine of these three fish were taken, ground, and mixed well separately. An amount of 6 mol/L hydrochloric acid was applied to hydrolyze the sample and then nitrogen was filled for 24 h. The prepared sample was used to detect the hydrolyzed amino acid contents by A200 amino acid analyzer with Aminosis.

Meanwhile, the flesh samples (0.1 g) were mixed with 0.3 mL water and 1.2 mL methanol, and then an ice-water bath was used to homogenize the samples with ultrasonic treatment for 10 min. After being frozen for 2 h at −20 °C, samples were centrifuged (12,000 rpm for 30 min at 4 °C) to collect the supernatant. Free amino acid analysis was performed by Waters ACQUITY Ultra Performance LC/MS (Waters, Milford, MA, USA).

### 2.7. RNA Extraction, cDNA Library Construction and RNA-seq at 2 Days

TRIzol Reagent (Takara, Dalian, China) was used to extract Total RNA from the liver in each groups according to the protocols. High-throughput sequence was conducted on 9 liver tissues collected from each group after 2 days of low-temperature stress (Figure 1). Among them, three fish from the same group (each fish 0.1 g liver) were mixed at random. Three biological replicates were put to use in the final RNA-seq. After that, the process of eukaryotic mRNA enrichment, first and second strand cDNA synthesis, adaptor, and sequencing on Illumina Hiseq4000 can all be referred to in our published paper [32]. Details of RNAseq reads and the total amount of RNAseq data of each individual sample can be found in Table S1.

### 2.8. De Novo Assembly, Functional Annotation, and Differentially Expressed Genes (DEGs) Analysis at 2 Days

Before the data were assembled, cut adapt software SeqPrep and Sickle (https://github.com/jstjohn/SeqPrep (accessed on 11 November 2021); https://github.com/najoshi/sickle (accessed on 11 November 2021)) was applied to filter unqualified raw data into clean data. The data processing process can also be referred to in published papers of our laboratory [32]. We assembled all the clean data using Trinity (https://github.com/trinityrnaseq/trinityrnaseq/wiki (accessed on 11 November 2021)) [33], then optimized and filtered the results by TransRate (http://hibberdlab.com/transrate/ (accessed on 11 November 2021)) [34] together with CD-HIT (http://weizhongli-lab.org/cd-hit/ (accessed on 11 November 2021)) [35]. The results, including 27,335 genes (G), 27,335 transcripts (T), N50 average length was 2653 bp. All transcripts obtained by this transcriptome sequencing were compared with GO and KEGG databases, and 19,305 GO annotations and 19,409 KEGG annotations were obtained. Details of transcripts and unigenes annotation are listed in Table S2. In order to compare different samples, the expression abundance of genes was quantified by using fragments-per-kilobase of exon model per million mapped reads (FPKM), measured by RSEM [36]. Given that the sequencing depth varied from samples, the FPKM value was used to normalize the absolute gene expression, making FPKM the expression quantity of genes. Then, we adopted DESeq2 [37] to verify the differentiation between StringTie assembled and quantified genes (|log2FC| > 1 was determined as the significant difference threshold, *p* < 0.05). Goatools (https://github.com/tanghaibao/GOatools (accessed on 11 November 2021)) [38] was applied for GO enrichment and KEGG pathway enrichment analysis.

### 2.9. Transcriptional Expression and Validation of Key DEGs at 2 Days

To verify the key genes expressions obtained from RNA-seq, RT-qPCR was performed. The process was set according to the previous operation of our laboratory [39]. We chose the gene B2M obtained by reference gene selection as the internal control. All the synthesis of primers in this study in Shanghai Generay Biotech Co., Ltd. (Shanghai, China), and the detailed information can be found in Table S3 and Document S1. On the basis of the manufacture’s protocol, SYBR Green (Takara, Dalian, China) was used for RT-PCR on Takara 800 Fast Real-Time PCR system.

### 2.10. Correlation Analysis

Pearson’s correlation test was conducted to figure out how the key genes correlated with SPSS 25.0.

### 2.11. Statistical Analysis

Data of crude fat content, plasma biochemical parameters index, hydrolysis amino acid, free amino acid content, and transcriptional expression were analyzed with one-way ANOVA by SPSS 25.0. The 2^−ΔΔCT^ method was applied to calculate the relative RNA expression, students’ *t*-test was applied to analyze the statistical difference by SPSS 25.0. In all figures, the statistical differences were represented as asterisks (*, *p* <0.05; **, *p* < 0.01; ***, *p* < 0.001) and results were expressed as mean ± SEM.

## 3. Results

### 3.1. Body Composition and Plasma Biochemical Parameters Induced by Hypothermia in A. grunniens

**Figure 2:**
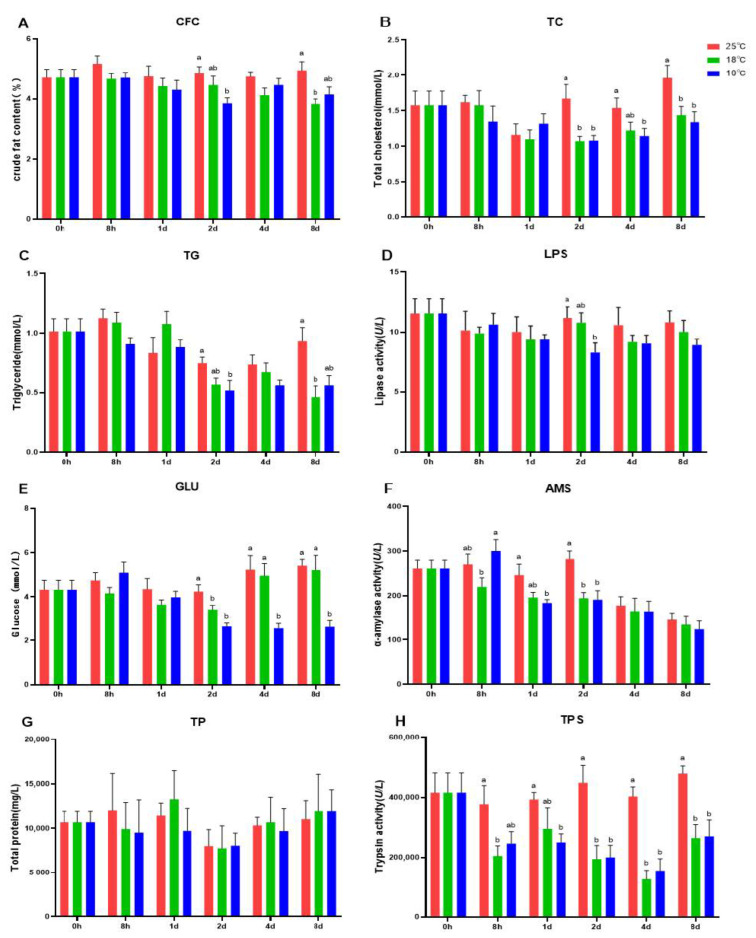
Body composition and plasma biochemical parameters of freshwater drums induced by cold stress (*n* = 9): (**A**) crude fat content, CFC; (**B**) total cholesterol, TC; (**C**) total triglyceride, TG; (**D**) lipase activity, LPS; (**E**) Glucose, GLU; (**F**) α-amylase activity, AMS; (**G**) total protein, TP; (**H**) trypsin activity, TPS. The statistical differences are indicated with different superscript letters (a, b) (*p* < 0.05).

Body composition together with plasma biochemical parameters induced by cold stress were first determined (shown in Figure 2). Results show that CFC (Figure 2A), TC (Figure 2B), TG (Figure 2C), and LPS (Figure 2D) in LT10 were decreased gradually in comparison with the Con (*p* < 0.05). Meanwhile, TC (Figure 2B) content in L10 was also dramatically decreased compared with Con after 4 days and 8 days stress (*p* < 0.05). Significant difference between LT18 and Con mainly occurred after 8 days (*p* < 0.05), including CFC (Figure 2A), TC (Figure 2B), and TG (Figure 2C). AMS decreased significantly (Figure 2F) after 2 days in LT10 and LT18 compared with Con (*p* < 0.05), and AMS in LT10 also showed a significant downward trend at 1 day (Figure 2F, *p* < 0.05), the downregulation difference of GLU persisted in the LT10 group at 4 and 8 days (Figure 2E, *p* < 0.05). Moreover, TPS in both LT10 and LT18 decreased significantly to about half of that in the Con group after 2 days (Figure 2H, *p* < 0.05). However, TP content showed no difference at each time point (Figure 2G, *p* > 0.05).

### 3.2. Amino Acid Contents Induced by Hypothermia in A. grunniens

On the basis of the results, hypothermia for 2 days induced significant alteration on crude fat content and plasma biochemical parameters relating to metabolism, which indicate that hypothermia for 2 days is a key time point to study metabolism regulation. Therefore, hypothermia for 2 days was selected for the subsequent analysis. We conducted the amino acid analysis at 2 days cold stress. From the results, hydrolysis amino acid content exhibited no significant differences among the groups under 2 days cold stress (Table 1). However, the free amino acid contents of arginine, histidine, lysine, methionine, phenylalanine, threonine, aspartic acid, glutamic acid, proline and tyrosine were decreased after low-temperature stress (*p* < 0.05). Meanwhile, the level of total essential free amino acid (EAA) decreased significantly in groups LT10 and LT18 (*p* < 0.05).

### 3.3. Transcriptome Profiling of DEGs Induced by Hypothermia in A. grunniens

**Figure 3:**
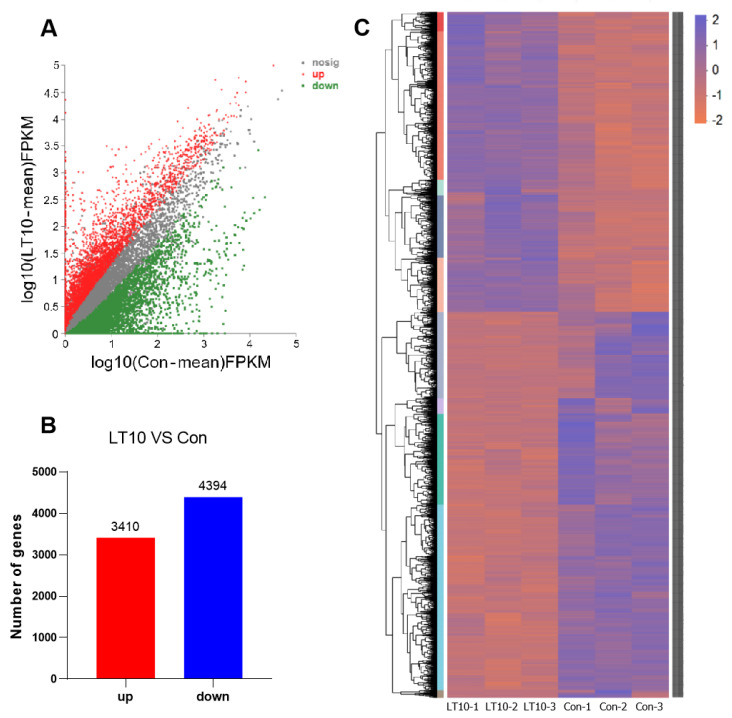
Transcriptome profile of freshwater drums liver induced by 2 days cold stress: (**A**) set diagram of DEGs; (**B**) up and down regulated DEGs; (**C**) heatmap of DEGs.

For the purpose of further exploring the inner mechanism of cold stress on freshwater drums, we conducted high throughput RNA sequencing to detect the DEGs between LT10 and Con. Taking |fold change| > 2.0 and *p* < 0.05 as threshold standard, 7804 DEGs in total was identified in LT10 (Figure 3A), including 4394 downregulated and 3410 upregulated DEGs (Figure 3B, Table S4). Since the genomes data of *A. grunniens* were not publicly available, most of the genes from transcriptome data were defined as those from *Larimichthys crocea*, which has an extremely high genetic similarity to *A. grunniens*. The expression levels and categories of DEGs demonstrated a pattern as clustering into sub-groups (Figure 3C).

### 3.4. GO and KEGG Enrichments of DEGs

The gene ontology as well as involved signaling pathways of DEGs were retrieved by subjecting them into the online database. The results were published in our previous research [23] (shown in Figure S1). DEGs were enriched into three kinds of items, including 6159 molecular functions (MF), 6648 cell components (CC), and 6826 biological processes (BP) (Figure S1A, Table S5). Analyzed by the rich factor value, most of the enriched items among the top 20 were related to biosynthetic and physiological metabolism, especially protein and amino acid metabolism (Amide biosynthetic process, 0043604; cellular nitrogen compound biosynthetic process, 0044271; cellular nitrogen compound metabolic process, 0034641; cellular protein metabolic process, 0044267; organonitrogen compound metabolic process, 1901564; peptide biosynthetic process, 0043043; peptide metabolic process, 0006518; protein metabolic process, 0019538; cellular amide metabolic process, 0043603) (Figure S1B, Table S6). Moreover, 342 KEGG signaling pathways were enriched by these DEGs, including 21 items with the corrected *p*-value < 0.05 (Figure S1C, Table S7). KEGG enrichment reveal that protein and amino acid metabolism (Pancreatic secretion, map04972; lysine degradation, map00310; tryptophan metabolism, map00380; histidine metabolism, map00340; protein digestion and absorption, map04974; valine, leucine and isoleucine degradation, map00280), lipid metabolism (Pancreatic secretion, map04972; linoleic acid metabolism, map00591; glycerolipid metabolism, map00561), as well as vitamin metabolism (Ascorbate and aldarate metabolism, map00053; retinol metabolism, map00830) were involved in the regulation of cold stress in freshwater drums. Specifically, PPARs signaling was dynamically enriched under hypothermia stress.

### 3.5. Expression of Lipid-Metabolism-Related Genes of A. grunniens under Low-Temperature Stress

**Figure 4:**
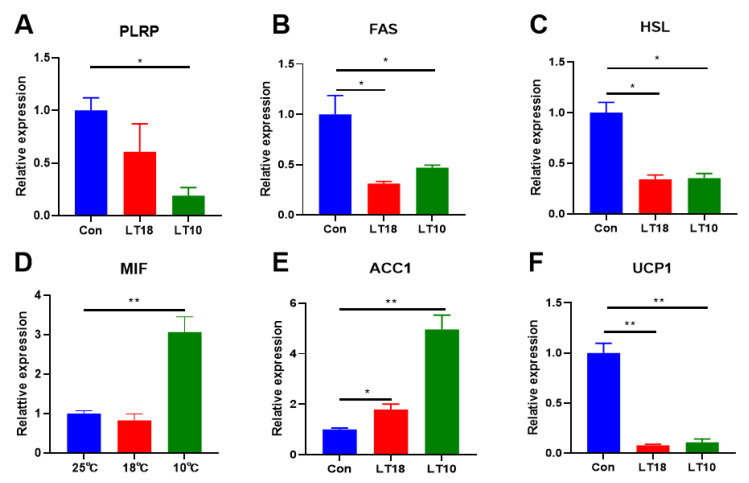
Expression of the lipid metabolism-related key genes in freshwater drums liver induced by 2 days cold stress (*n* = 9): (**A**) pancreatic lipase-related proteins, PLRP; (**B**) fatty acid synthesis, FAS; (**C**) hormone-sensitive lipase, HSL; (**D**) cytokine macrophage migration inhibitory factor, MIF; (**E**) acetyl Coenzyme A Carboxylase 1, ACC1; (**F**) uncoupling protein 1, UCP1. Asterisk represents the statistical difference (*, *p* < 0.05; **, *p* < 0.01).

**Figure 5:**
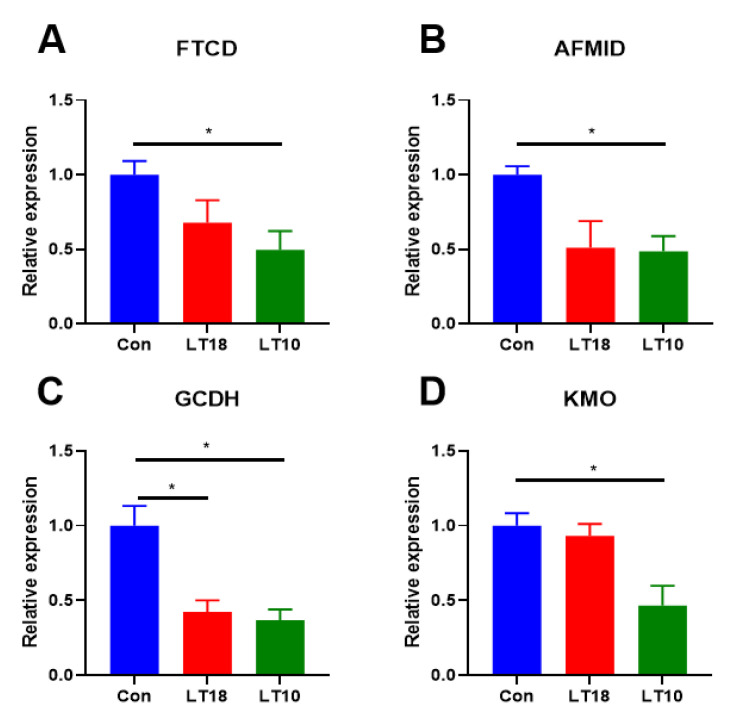
Expression of the amino acid metabolism-related key genes in freshwater drums liver induced by 2 days cold stress (*n* = 9): (**A**) formiminotransferase cyclodeaminase, FTCD; (**B**) enzyme arylformamidase, AFMID; (**C**) glutaryl-CoA dehydrogenase, GCDH; (**D**) kynurenine3- monooxygenase, KMO. Asterisk represents the statistical difference (*, *p* <0.05).

DEGs involved in lipid metabolism were first identified and the transcriptional expression were validated with RT-PCR (shown in Figure 4). Results shown that the expression of pancreatic lipase-related proteins (PLRP, Figure 4A), fatty acid synthesis (FAS, Figure 4B), hormone-sensitive lipase (HSL, Figure 4C), acetyl coenzyme A carboxylase 1 (ACC1, Figure 4E), and uncoupling protein 1 (UCP1, Figure 4F) was dramatically decreased (*p* < 0.05). Conversely, the expression of cytokine macrophage migration inhibitory factor (MIF, Figure 5D) was increased significantly (*p* < 0.05).

### 3.6. Expression of Amino Acid Metabolism-Related Genes of A. grunniens under Low-Temperature Stress

KEGG enrichment revealed that amino acid metabolism was active to the regulation of cold stress in freshwater drums (Figure 5). Results indicate that the transcriptional expression of formiminotransferase cyclodeaminase (FTCD, Figure 5A), enzyme arylformamidase (AFMID, Figure 5B), mitochondrial enzyme glutaryl- coenzyme A dehydrogenase (GCDH, Figure 5C), kynurenine3-monooxygenase (KMO, Figure 5D) was significantly downregulated (*p* < 0.05).

### 3.7. PPARs Signaling Was Involved in the Regulation of Hypothermia in A. grunniens

**Figure 6:**
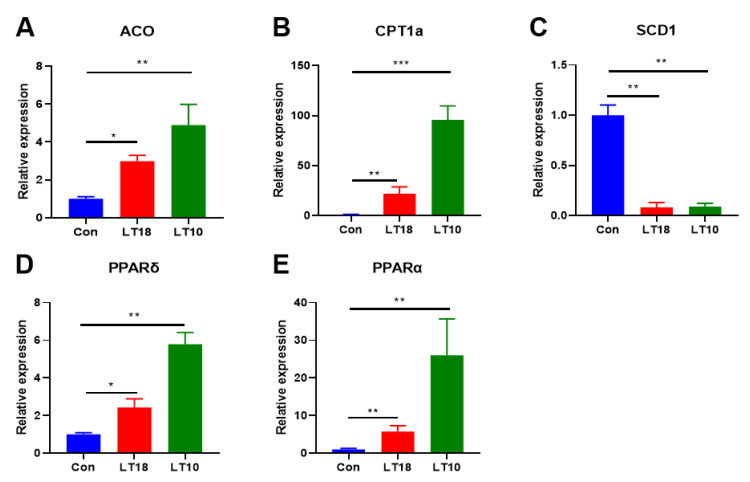
Expression of PPARs signaling related genes in Freshwater drums liver induced by 2 days cold stress (*n* = 9): (**A**) acyl-coenzyme A oxidase, ACO; (**B**) carnitine palmityl -transferase 1A, CPT1a; (**C**) stearoylcoa desaturase-1, SCD1; (**D**) PPAR delta, PPARδ; (**E**) PPAR alpha, PPARα. Asterisk represents the statistical difference (*, *p* < 0.05; **, *p* < 0.01; ***, *p* < 0.001).

Apart from the above DEGs related to lipid and amino acid metabolism, the transcriptional expression of PPARs signaling related genes (shown in Figure 6), which is closely associated with metabolism homeostasis, was also validated. Results indicate that acyl-coenzyme A oxidase (ACO, Figure 6A), carnitine palmityltransferase 1A (CPT1a, Figure 6B), PPARδ (Figure 6D), and PPARα (Figure 6E) were dramatically upregulated (*p* < 0.05), but stearoylcoa desaturase-1 (SCD1, Figure 6C) was downregulated significantly (*p* < 0.05).

### 3.8. Lipid and Amino Acid Metabolism of A. grunniens Were Co-Related with PPARs Signaling under Low-Temperature Stress

**Figure 7:**
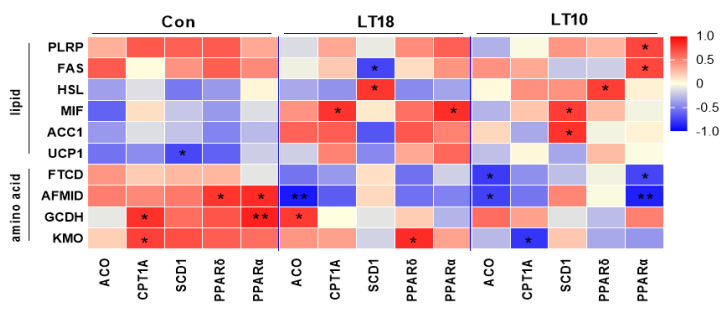
The metabolism of lipid and amino acid were co-related with PPARs signaling under hypothermia in freshwater drum. Asterisk represents the statistical difference (*, *p* < 0.05; **, *p* < 0.01).

According to the aforesaid data, Pearson correlation analysis was proceeded in order to reveal the relationship between PPAR pathway and lipid and amino acid metabolism. The results shown that PPAR signaling was positively correlated with lipid metabolism in group LT10, while the correlation between PPAR signaling and amino acid metabolism changed from positive correlation at 25 °C to negative correlation at 10 °C (Figure 7).

## 4. Discussion

Generally, the growth of fish requires a relatively stable water temperature. However, extreme low or high temperature stress caused by abnormal weather changes generally exists in the practical aquaculture production [40,41]. The aim of our research is to assess the impact of low water temperature stress on the physiological and metabolic alternation of freshwater drums.

As a complex metabolic transformation process, fish growth includes the utilization of glucose, amino acids and fatty acids, intracellular protein transformation, as well as fat deposition, together with the regulation of hormones and other nutrients, leading to the accumulation of lipid and muscle [42]. To combat the low-temperature environment, aquatic animals have evolved to adapt to adverse stress, such as to enhance glycol-metabolism and decrease food intake. Cold stress experiments in tilapia showed that plasma glucose increased immediately, and then decreased after 2 days stress [10]. Decreased glucose may lead to insufficient energy supply and affect other physiological functions of the body, such as the previously reported reduction of immune and antioxidant properties [23]. Therefore, it will have adverse effects on the health of the fish. In our experiment, glucose, lipid, and amino acid metabolic-related enzyme activity was decreased along with the temperature decreasing. The results indicated that low temperature may reduce the digestive enzyme activity, and thus affect the physiological metabolism of freshwater drums.

Protein is an important nutrient for aquatic animals. Cold stress also affects the decomposition and anabolism of protein. Cold stress affects biological enzyme activity, reduces, or hinders cell metabolism and protein synthesis [11]. Studies on *Perccottus glenii* showed that the composition of free amino acids in different tissues changed under long-term cold stress [43]. In our present study, trypsin was decreased sharply under hypothermia stress, indicating that the protein digestive activity was decreased in freshwater drum [44,45,46]. Analogously, free amino acids (FAA) content was also decreased, especially the content of free essential amino acids (EAA). This result is consistent with the results of low-temperature stress experiments on *Litopenaeus vannamei* [47].

These different phenotypes in body composition, plasma biochemical parameters, and free amino acid contents positively indicate that cold stress could affect the physiological metabolism of freshwater drums. However, the underlying mechanism remain unclear. With RNA-seq, we found the DEGs were mostly enriched in amino acid metabolism and lipid synthesis and digestion. This shows that although freshwater drums can tolerate low temperatures for even 10 °C, its metabolism will still be affected when the ambient temperature drops.

Hypothermia may enhance lipid metabolism in fish by decreasing plasma TG content together with upregulating the expression of genes related to lipid metabolism [48]. In this study, low-temperature stress downregulated the expression of FAS, HSL, PLRP, ACC1, and UCP1, which function to catalyze the synthesis of fatty acids [49], catalyzes the hydrolysis of triacylglycerol [50], and lipid digestion [51], de novo fatty acid synthesis [52], and energy supply by fatty acid catabolism [53], respectively. Additionally, metabolic and inflammatory related MIF was upregulated under hypothermia, indicating that metabolic alternation was corelated with inflammatory response in freshwater drum [54]. These data reveal that fat synthesis and metabolism might contribute to the immune resistance under hypothermia.

In addition, amino acid metabolism-related gene expression was also validated. FTCD encodes an intermediate metabolic enzyme that links histidine catabolism with folate metabolism [55]. AFMID is involved in tryptophan transport [56]. KMO plays a central role in tryptophan metabolism [57]. GCDH can affect the accumulation of glutaric acid as well as 3-hydroxyglutaric acid in the catabolic metabolism of tryptophan, lysine, and hydroxylysine [58]. In this experiment, the expression of AFMID, KMO, and GCDH were all downregulated after the cold stress, indicating that hypothermia inhibited the amino acid metabolism of freshwater drums.

PPARα regulates the expression of ACO and CPT1a by inducing the oxidation rate of mitochondrial and peroxisomal fatty acids [59]. SCD1, a target of PPARα, is the key enzyme in the biosynthesis of monounsaturated fatty acids [60]. In our experiment, ACO, CPT1a, PPARα, and PPARδ were upregulated while SCD1 was downregulated under hypothermia, which was consistent with the study on *Larimichthys Crocea* [61]. Additionally, as a possible biomarker, glycine may be able to evaluate lipid accumulation as well as the lipid-lowering effects of PPARA/G in oleate-treated macrophages [21]. The activation of the PPAR pathway can also inhibit insulin sensitivity, thus could reduce glucose content [62], which was also confirmed in our study, evidenced by the decreased glucose and upregulated PPAR related genes. In this study, the relationship between PPAR signaling and amino acid metabolism and lipid metabolism was also validated, the results showed that there is a reversal of the correlation between PPAR pathway and fat metabolism and amino acid metabolism after 10 °C cold stresses, which indicated that the PPAR pathway maintained the homeostasis of lipid and amino acid metabolism in freshwater drums at low temperature.

## 5. Conclusions

In this study, hypothermia inhibited lipid and amino acid metabolism by the digestive enzyme activity, lipid and amino acid synthesis, and catabiosis. Transcriptome and RT-PCR analysis revealed that hypothermia-derived DEGs were chiefly involved in lipid and amino acid metabolism Moreover, PPAR signaling was dynamically related to lipid and amino acid metabolic homeostasis under low-temperature stress. These results uncovered the molecular basis of hypothermia on freshwater drum and provided potential regulation in resistance to hypothermia by means of metabolic approaches.

## Figures and Tables

**Table 1:** Amino acid content (dry weight) after 2 days temperature stress (g/100 g sample). Note: values are mean and SEM, *n* = 3. Values in the same line with different superscripts are significantly different (*p* < 0.05). Different superscript letters (a, b, c) represent the statistical difference (*p* < 0.05).

Amino Acids	Hydrolyzed Amino Acid			Free Amino Acid		
	25 °C	18 °C	10 °C	25 °C	18 °C	10 °C
Arginine	2.61 ± 0.08 ^a^	2.95 ± 0.13 ^a^	2.85 ± 0.29 ^a^	0.0157 ± 0.00639 ^a^	0.005 ± 0.00298 ^b^	0.0024 ± 0.00017 ^b^
Histidine	1.00 ± 0.04 ^a^	0.92 ± 0.05 ^a^	0.90 ± 0.10 ^a^	0.1236 ± 0.01007 ^a^	0.0107 ± 0.01044 ^b^	0.0348 ± 0.01737 ^b^
Isoleucine	1.8- ± 0.02 ^a^	2.07 ± 0.12 ^a^	2.00 ± 0.23 ^a^	0.0062 ± 0.00316 ^a^	0.0045 ± 0.00066 ^a^	0.0044 ± 0.00031 ^a^
Leucine	2.91 ± 0.04 ^a^	3.34 ± 0.18 ^a^	3.21 ± 0.36 ^a^	0.0168 ± 0.00251 ^a^	0.0095 ± 0.00135 ^a^	0.0051 ± 0.00252 ^a^
Lysine	3.48 ± 0.08 ^a^	3.78 ± 0.20 ^a^	3.80 ± 0.43 ^a^	0.1586 ± 0.01514 ^a^	0.0543 ± 0.01039 ^b^	0.1242 ± 0.01722 ^ab^
Methionine	0.85 ± 0.05 ^a^	0.87 ± 0.07 ^a^	0.82 ± 0.09 ^a^	0.0074 ± 0.00178 ^a^	0.0161 ± 0.00744 ^a^	0.0178 ± 0.01332 ^a^
Phenylalanine	1.65 ± 0.02 ^a^	1.84 ± 0.09 ^a^	1.78 ± 0.19 ^a^	0.0084 ± 0.00149 ^a^	0.0043 ± 0.00118 ^b^	0.0041 ± 0.00029 ^b^
Threonine	1.36 ± 0.02 ^a^	1.53 ± 0.07 ^a^	1.46 ± 0.15 ^a^	0.0681 ± 0.00878 ^a^	0.0344 ± 0.00307 ^b^	0.0427 ± 0.00361 ^b^
Valine	2.08 ± 0.02 ^a^	2.32 ± 0.12 ^a^	2.25 ± 0.25 ^a^	0.0428 ± 0.00213 ^a^	0.0215 ± 0.00957 ^a^	0.0227 ± 0.00926 ^a^
total EAA	17.73 ± 0.33 ^a^	19.62 ± 1.02 ^a^	19.07 ± 3.62 ^a^	0.4476 ± 0.00548 ^a^	0.1603 ± 0.01095 ^c^	0.2581 ± 0.02536 ^b^
Alanine	2.86 ± 0.09 ^a^	3.13 ± 0.12 ^a^	3.05 ± 0.26 ^a^	0.082 ± 0.00739 ^a^	0.0541 ± 0.02722 ^a^	0.0681 ± 0.00964 ^a^
Aspartic acid	4.19 ± 0.07 ^a^	4.73 ± 0.39 ^a^	4.57 ± 0.48 ^a^	0.0288 ± 0.00435 ^a^	0.0166 ± 0.00088 ^b^	0.0203 ± 0.00239 ^ab^
Cysteine	0.05 ± 0.00 ^a^	0.06 ± 0.01 ^a^	0.05 ± 0.01 ^a^	0.0006 ± 0.00019 ^a^	0.0006 ± 0.00035 ^a^	0.0003 ± 0.00009 ^a^
Glutamic acid	6.49 ± 0.11 ^a^	7.20 ± 0.35 ^a^	7.01 ± 0.73 ^a^	0.12757 ± 0.01672 ^a^	0.06913 ± 0.00636 ^b^	0.08979 ± 0.00538 ^ab^
Glycine	3.97 ± 0.23 ^a^	4.16 ± 0.09 ^a^	4.12 ± 0.35 ^a^	0.3315 ± 0.02239 ^a^	0.2898 ± 0.02433 ^a^	0.329 ± 0.05891 ^a^
Proline	2.20 ± 0.09 ^a^	2.31 ± 0.06 ^a^	2.40 ± 0.26 ^a^	0.0899 ± 0.01459 ^a^	0.0346 ± 0.01009 ^b^	0.053 ± 0.00871 ^ab^
Serine	1.46 ± 0.03 ^a^	1.65 ± 0.07 ^a^	1.57 ± 0.17 ^a^	0.0051 ± 0.00199 ^a^	0.0112 ± 0.00702 ^a^	0.0043 ± 0.00183 ^a^
Tyrosine	0.93 ± 0.03 ^a^	1.14 ± 0.06 ^a^	1.03 ± 0.12 ^a^	0.0075 ± 0.00165 ^a^	0.0014 ± 0.00127 ^b^	0.0003 ± 0.00006 ^b^
Total NEAA	22.04 ± 0.59 ^a^	24.39 ± 1.67 ^a^	23.78 ± 2.37 ^a^	0.6730 ± 0.06312 ^a^	0.4692 ± 0.06114 ^a^	0.5652 ± 0.07076 ^a^

## Data Availability

The data that support the findings of this study are available from the corresponding author upon reasonable request. Data is not publicly available due to privacy.

## Supplementary Materials

The following supporting information can be downloaded at: https://www.mdpi.com/article/10.3390/metabo13010102/s1, Table S1: Transcriptome sequencing mapping statics; Table S2: Transcripts and unigenes annotation; Table S3: Primers and sequences referred to in the experiment; Table S4: Statistical analysis of express; Table S5: GO classification table of gene; Table S6: GO enrichment analysis; Table S7: KEGG enrichment analysis; Document S1: cDNA sequence of the gene involved in the experiment; Figure S1: GO and KEGG enrichments of DEGs in Freshwater drums liver induced by 2 days cold stress.
