# Supplementary material for: PPAR Signaling Maintains Metabolic Homeostasis under Hypothermia in Freshwater Drum (Aplodinotus grunniens)
Source: Metabolites. 2023 Jan 8;13(1):102. doi: 10.3390/metabo13010102 (PMC9865675; doi:10.3390/metabo13010102)
Supplement: Supplementary file 1 [file metabolites-13-00102-s001.zip › Document S1 cDNA sequence of the gene involved in the experiment.pdf]

FTCD

gAGTCTGCAGACACTGCCATGGCTCGGCTGGTGGAGTGTGTCCCAAACTTCTCCGAGGGTCGAAGTA
AAGAGGTGATCGATGCCATCTCTGCAGCCATATCTGGCACCTCTGGCTGCAGCCTGCTGGATGTCGA
CCCCGGAGCCTCCACCAACCGGACCGTCTACACCTTCGTGGGCTCTCCGGAGGCCGTGGTGGAGGGA
GCGCTGAATGCTGCCCGGCAGGCCTTCAGCCTCATCGACATGAGCAAACACTCAGGTGAGCATCCTC
GGACTGGAGCGCTGGATGTTTGTCCCTTCATCCCCGTCCAGAACGTCTCCATGGACGACTGTGTCAAG
TGCGCCAACATGTTTGGACAAAAACTCTCTGAAATGCTGCACGTCCCCGTGTATCTTTACGGAGAAG
CAGCGCGGAAAGAAACAAGGAGAAATCTTCCATCCGTCCGTGCCGGAGAGTACGAAGCTTTACCTG
AGAAGTTGAAGCGTGATGATTGGGCGCCAGACTTCGGCCCCGCCTTGTTCGTACCGTCATGGGGCGC
CACAGTAACCGGCGCGCGAAAGTTCCTGATCGCGTACAACGTGAACCTGATCAGCACCAAAGAACA
GGCTCATCGCATCGCTCTGGACGTCAGGGAGCAGGGCCGCGGCAAAGACCAGCCCGGGCTGCTGCA
GAAGGTGCAGGGCATGGGCTGGTATCTGGACGAGGCCAACATCGCTCAGGTGTCCACCAACATCCTG
GACTACGAGCTGACCCCCCTCCACACCGTGTACGAGGAGATCTGCAGGGACGCTGAGGACCTGAAG
CTGCCTGTGGTCGGCTCTCAGATCGTCGGTCTGATCCCTCTGAAGGCGCTGCTGGACGCTGCTGACTT
CTACATCCACAGAGACGGACTGTTCGTTGTGGAGGAGGAGCACAAAATCCGGCTGGTGATCAGCAA
ACTCGGCCTCGACTCTCTTGGTCCGTTCAACCCGAAGGAGAGAATCATCGAGTACATGGTGAGGTCA
CAGGAGGACAGTGGGCTCGTGTCTTTGCCTCTGCAGCAGTTTGTCCGGAGCGTTGGATCTCGGACAG
CGGCGCCCGGTGGAGGATCTGTTTCTGCTGCCATTGCTGCTCTGGGGGCGGCATTGGGCGCCATGGT
GGGTCAGATGACGTATGGGAAGAGGCAGTTTGCGAACCTGGATGGGGTAATGAGGAGGCTGATTCC
TCCGTTTCATCAGGCCATGAACGAGCTGCTGCTCATGGTGGACGCCGACTCGTCCGCCTTCAACAGCT
ACATGGCGGCACTGAAAATGCCAAAAAACTCAGCAGAGGAAATCAAAAGGCGACAGGCTGCGATGC
AGGAAGGTCTGCAGGGGGCGGTGGGCGTCCCTCTTGCTCTGGCTGAGAGGATCAGTGTCCTCTGGCC
GTACCTTAAAGAAATGGTCGCCTACGGAAACATCGCCTGCAAGTCAGACGCTCAGGTGGCAGCTAAA
GCTTTGGAGACGGCTGTTTTCGGTGCGTATTACAACGTCACCATCAACCTCAAAGACATCGTGGACG
ACGCCTTCAAGACGGCTACTCAGTCGAGAGCGTCGGCGTTGCTGCAGGAAGCGAAGGAGAGCGCTG
CCGCCATCCTCCACGCTGCTGAGGAGAGAAACTGAAGGCAGAGACTTCCACAGTCTGACGCTGCTCT
GTGACTGGATGTTTTCTTGCAGCAACGCACTCTGGGACCAGTAGTTGACTAAACCAGATTTAAGTTTA
TGGAACAATAATAATTTCTGTTGATTGTAAATATTCATAATGGAAAATAAATGTTGACACAAGAGTG
ACCAAATAAACAGATTTACTGTATGAAGCTGTCTGAAGTCTGGACCAGCAGAGTCCTGGACCAGGTT
TATTAAAGAAGGTCTAATAAAGTTTATAAAGTCTCTTTAg

AFMID

gAGAGTAGACTGGATTTTACTGTGGTATCGGGTGGATACATCTGTGATCGGACCTTCAACGAGTTAAA
TCACTTCAAGACATAAAACGCCGCACTGGACTGAAATGAATGAGCTCGAGAGGCAGTACTCACCCA
GCCGGTGGTCGCACAGGATGTCGTCGGACGACGTGATCAAAGCTCATGTGAAGGCTTTGAAAGAAG
GTACCGAGCGTGCCCGTGGTCTGGCTCAAACCTTGCTCAACGTGCCATACGGGGAGGGAGATGGAGA
GAAACTGGATATCTACATACCCAGCACCAGCTCTTTAGATGTCAATCTTGTCATTTACCTACATGGAG
GCTACTGGCAGTTCCTCAGCAAGGAGGAGTCAGGATTCATGGCTGTTCCACTCGTTGATAAAGGTGT
AGTGGTGGTTGCCGTTGGCTATGACATCGCCCCCAAAGGTAACATGGATCTGATGGTGTCTCAAGTA
CGCAGGAGTGTTGTGTCTGTTGTTCAGCAGTATTCTCACATCAGTGGTCTGTACCTCTGTGGCCATTC
TGCTGGGGCTCACCTGGCTGCAATGGTCCTCTCCACTGACTGGTCGCAGTATAGTATCACTCCTCAGA
TCAAAGGTGCTTTCCTTGTCAGTGGCATATATGACCTCCTGCCCATCCTGTCCACCTACGTCAACGAG
CCTTTGAAGATGACAGAGGAGGTGGCAGTGAGGAACAGTCCCAGCAAGCTGGTCCCTCAGCTCAAA
CTCTCCTCCTCCAACTGTGACATCATTGTGGCCGTTGCCGAGAACGACTCGCCAGAGTTTCGTAAGCA
GTCGGAAGAATACTACAAAACTTTGCAGGCATCAGGACTTAATGTGACCATGGTGGATGTGGCGAAC
ACAGACCACTTCAGTATCACTGAGCAACTGGTAGATGGGGAGTATCACCTAACGAAGCTTCTCTTGA
AGATGATGGGTAAGAGCTGAGGCAGCAATCCTTGATCCGTCATCTGATATCAACATACCTCTGTTTC
AACATACACCTCTAAACAGAGTCATAGTGCACCGATACTGATACAATATGCATTATTTCAGGTTTGTA
TGATGGTAAATGTACAATTACTGTCCACTGTGATTGATCAACAATAATGATGGACTGTTTCATTAGTT
ATATCTCTGTTCAGTGACATTATCATATTATGAAAACATTATGTGCCTGAAACCGAGTCACTTCAATG
ATTTTGACTGCAAATTATACTACAGTTAAACCTGCTTAGTTGTAAAGAGATTATTTAACAACCATTGT
GCACAATGTTGTTGTCAGGAAATGCTGTTCCACTGACATACTGAGAGTTGTTGAGCTAATGGTTAGTT
TTGGCATATTTCCAGTGTTTTGTAAAAGGGTGGGTCTGTCTGTTTAGTGTTTTTTGGTTGTTTTTTTTTT
TTCTTTCCTGTGACAATCTGCACCACAACTTTAAGTCCATGGGTAGGACAAGAATCATGTAAAGAGT
ACTGATAATACAAAGCGAATGTAGCTTTTTATTGTAACCAGCAGTAAACTTTAATGTGTTGTCCACGC
TTGATAAATGTTGCTAATAAAAACTATATATTTGCACTTCTATCCTAATGCAGTAGAAGAATACAGAT
ATTCAAATGCACATATTTTTAAAGAAAAATGGGACTAAATTTAGGTGATGCACATTGTTACTTACTGC
CCTCAGTCTAGCATATTAAATAATTGTTTACATTATTGTTTACACAGTGTTCTATTATTAACCTGCAAC
AACTGTAGATTATAAAGAGAATCGTCACAATAAATATTTATATTTGTGAgtttctgtg

KMO

gACTTTGACCTGGACAGTGAGGAGGGCCAGACCACCAGAGAGCTGCCAGCACAATGGAGCAAATGA
CCAGCAGATCAAACAAGAAAAAAGTAGCAGTGGTGGGCGGTGGTTTGGTTGGAGCACTGAATGCCT
GCTTCTTTGCCAAAAGAGGCTTTGATGTGGAAGTGTTTGAAACCCGTGAAGATATCCGGCAAGCCAA
AATTGTGAAGGGGAGAAGCATCAACCTGGCTTTGTCTCACCGAGGCCGGCAAGCACTGAAGCATGTT
GGAATGGAGGAGAAGATTGTCTCTCAGGGAATCCCCATGCACGCAAGAATGATTCATTCCCAGAATG
GGAAACAATCTCCGATTCCTTATGGCAAGAAAGGCCAGTACATCCTGTCAGTAGACCGAGCCAATCT
AAACAAACAGCTGCTAACAGCGGCAGAGACATATCCAAACACAAAGTTGAACTTTGACTACAAACT
GCAGGACTGGAGTGCTGAAACAGGGCAAATGACCTTTGTCAGGTCAGATGGCTTAGAGGAGCAGAT
CAAGGCAGACCTGATCGTAGGCTGTGATGGAGCTTTCTCTGCCATCCGCAAGCAATTCCTTCGTCGCA
GCCGCTTCAACTATAGCCAGACCTACATCCCCCATGGCTACATGGAGCTCACCATGCCACCTATAAA
TGGAGAGTTTGCCATGAAGCCTAATTATCTGCACATCTGGCCACGAAACACATTCATGATGATCGCC
CTGCCCAATTTGGACAAGACATTTACCTGTACCCTCTTCATGCCCTTTGACGAGTTTGAGAAGATCAC
CACCGGAGATGAAGTCATCGAGTTTTTCCAGAAATACTTCCCTGATGCCATACCACTAATAGGAGTT
GATAGTCTCAAGAGGGATTATTTCCGATTGCCTGCGCAGGCCATGGTGTCTGTCAAGTGCACCCCAT
ATCACATCGGTGACAAATGCGTCCTTATGGGCGATGCTGCCCATGCAGTGGTTCCTTTCTATGGGCAG
GGGATGAACGCTGGCTTTGAGGACTGCATCGTCTTTGAAGAAGTAATGGAACAGTTCAATGAGGATT
TCAGCATTGTTCTGCCTGAGTACACCAGAGTGCGAGTTCCAGACGACCATGCAATTGCAGACCTGGC
TATGTACAACTACGTTGAAATGCGAGCCCATGTCAACTCCAGATGGTTCCTTTTCAGAAAACATGTCG
ATAACTTCCTCCACTTCCTCATGCCAAAGACAATAATTCCACTTTACACAATGGTCACTTTCACCAGG
ACGAGGTACCATGAGTGTGTGGAGCGTTGGCAATGGCAAAACAAAGTCATAAACCGTGGCCTGCTG
GTCAGTGCTACAGGATTGGTTTTGGGAGGATCCTACCTGCTCATTAAAAATCCTCCTGATATCAACAA
ACTCATCATCCCTGCTGAGAAAATGTGGAATAGGCTCATGGCCCTGAGGACCCCATGAGCGCTGATG
CTGTCTCTGATCTGGCAGGCTAATGCAAAGATTGGAGCACCAACCACTAGAAATGAACTCAGAGGGT
GCAAATTTTAATGAGTGATTTGATTGAATCAATCAATGAAAGCTCTGCCAGGCAAATTAATTTATGAT
TATAATCATTGCCATAATGTATACCACTCATAATGATTGATAATATTATATTAGCGTTTTACTGATAA
TGGTGTGACTGCAGGAGTGCAACAGATTTGAGGCTTTTCATGCCATCATTATTTTTAAAGACAAAAAT
GCCAGTATTAAACTCTCAGGCAGGGGCTAGGGTACATCCTGTTTAAGTTTACACTTTTACATGAATTG
CAAATAAATGTTCCCTCAGGTCATAGTTACTTTTTTTATTTTGCCTTAAGGATTCTTAAAACGAGGAA
CATTTTGGGACCATATCAACTCTGGATCAACAAATTCTTAAACCTGATTTTTTTTTTTTTTGGCCATT
TGGGGGCAGCAGAAACAAGTTGCCAAAAATAGCTTCCAGTAGGCAGTTATCTTAGTTTTGCATAAAA
ACTGGAAACAGTTAGCATGGCTTTCAAAAGTTAAAAGACATATCCGGGTAGCAGAACGTTAACTAAT
TAACACATTATATCTCATTTGTTTACTCCGTCTATAAAACTGTCTATAAAACTGGAGAGT

GCDH

ggtgATATTTTTATGTTTCATTCTCACAATGTACACATGCTGCCGCTGTACAGACAACATGTTTGACTTT
GCACGAGAACAGCTGATCACGGTTTAATATTTGCCGCGTGCTTTTATGATTACGCCACATTCGATATG
ACACGCAGCTCGGTGTGCTCTACGTGATGACGTCAGGCCGTCCAGTAGCTGCGCCGTCAACCCGAGA
GGCAGTAAAGGGGAACCCGGTGCTCCCGTTAACGCAGAGCAGTCTTCATCATGGCATTGAGAGGCGC
CTTGTGCCGTCTCCTGGTCAGACCTCAGAGATGTGCCGTCATCTCTGCATGCAGGGCACAGGGAACC
GCAGCACCTGTCAGGAGGGATGTAGAGAAGACCGAGCAGAAAGCTAAAGCACCGAAGGTCCAGTTC
AACTGGCTTGATGCCCTGGACCTGGAGGGCCAGCTGACAGAGGATGAGATCATGATCAGAGACTCCT
TCCGGACCTACTGCCAAGAGAAACTCATGCCTCGCATCATCATGGCAAACAGAAATGAGGTGTTCCA
CAGAGAAATTGTGTCAGAGATGGGAGAGATGGGTGTCTTGGGCCCGACCATTAAAGGTTATGGCTGT
GCTGGGACAAGCTATGTGGCCTATGGTCTGATTGCCAGAGAAGTAGAGAGAGTGGACAGTGGCTATC
GATCTGTCATGAGCGTGCAGTCGTCACTGGTCATGCACCCCATTAACGCCTATGGCACAGAAGAGCA
GAAACAGAAGTACCTCCCCAAGCTGGCTCGTGGAGAGATCCTGGGTTGCTTCGGCTTGACAGAGCCT
AACCATGGCAGCGACCCCGGCAGCATGGAAACCAAAGCCAAATACAATCCATCCAGTCGCACCTAC
AGTCTGACTGGCTCGAAGACCTGGATCACTAACTCCCCGGAGGCAGATATTGCTGTTGTGTGGGCCA
AATGTGATGACGGGAAGATCCGCGGCTTTATCTTGGAGCGCGGCATGAAGGGCCTCTCAACACCTAA
GATCGAGGGAAAGTTCTCCCTAAGAGCCTCCGCCACCGGTATGATCATCATGGACGAGGTGGAGGTC
CCTGAGGAGAACCTGCTGCCTAAAGCCAGCGGCCTCGGCGGTCCTTTTGGCTGCCTGAACAATGCTC
GTTATGGGATTGCGTGGGGCGCTCTGGGTGCTGCAGAGTTCTGTTTCCACACAGCTCGACAGTACAC
ACTCGACAGAATCCAGTTTGGCGTTCCACTGGCAAGAAATCAGCTGATGCAGAAGAAAATGGCCGA
CATGTTGACAGAGATCACCATCGGCCTGCAGTCCTGTCTGCAGCTGGGAAGACTCATCGATGAGAAA
AAAGCAGCCCCAGAGATGATATCCATGCTGAAGAGGAACAGCTGCGGGAAAGCCCTTGACATCGCC
AGGCAGGCCAGAGACATGCTTGGAGGAAATGGCATCGCAGATGAGTACCACATCATCCGCCACGTC
ATGAACCTAGAAGCTGTCAACACATACGAAGGTACTCATGACATCCACGCCCTGATCCTGGGCAGAG
CCATCACAGGGCTGCAGTCCTTCACCGTGGACAAATAGACCCCGCCTCTCACTGCTGCTCCCGAGGA
ACCTGCTGTTAAAAATGTAACTCGCATGTCATCTGTGCAGCAGTTCTCACTTTATTTGAACAGATTCT
TACCTGTATACTGACAATGCATGGTTTGTCTCAGGTTTATATATTTGAGTGAAGAAGTCACGTTTGAG
TCTGAACTTGTGGACTGTTGTGACGTGTAATTAATTTAGAAAAAGATGTTTTCATAAAAAGGGCTATT
TTTCTACTCAATCAAAGCTTTTCAGCTCTGTTTCCCAGTTGTATAGACAAAGTAAAATTCATTGTATA
GGCTGACGGACACTACTGTAGTGAAGCATTAACAGTCCTGAATAATGCTGCTGGTTACTGATGTATG
CAACATTAATACTGTCATTATAAGAGAACAAAGCATAAATGGCAAGAACCTCTTTGGGATTTTCCGT
GCAACTTTTTTGTTTTTAAGTAGATCAAATCTCTGTTATGTGATAACATTGTTGGCCAGAGGTCAAGG
ATGTTGATCAGAATAAAAGTTATTTAAGAGTTTCAAATTGCTGCAGCAGAATAAAGTCTAAGTTTGA
AAAGGT

PPAR δ

atgTGACGATGGAGGAGGAGCGGTGGGGAGTGAGTGAGGCAAAGCAAAGCAAAGCTAGGAGCAACAAG
GAGAGTCGGGGAGGGATTATCAGTTAACTGTGCTTATCTCCATGTGATATGGTGGCGAACAAAAGTGTCCCG
ACTTGCTTTTCTTTTTTCTCAGCTCGGTCGCTCACATGAAGGCCTGAACCTAATCTTTAAGTTTATGTTAGCGG
TCTGACGTTAGCTCCCGCTGCTGGTGCTGCTGGTGACTTCAAACAACTTGTCAACAGCCGATCGATCCTGGA
CGGGGATTTTTTTTTTTTAGGAGTTGTCTGCCACTTGTGTAATTTTGAGCCTGTTGGCAGCTTCTCACAGCGT
AAACATCAACCAAGCAGCACTATTTGGGAGAGTGGGGCTATGATGGCAACGTGACAGGAGGGGAGCCGCT
GAGTCAGCTTCAACAGGAACCATGGAACGGCTTCAACAAACTGCTACAGAGCAGCACGACAGGGTGAACG
GCTACTGCGAGCCCAAATCCCCCCAGGACACGGCCGATGTCAGGTGGAAGGCGCTGGAGGGAGAGTCCG
GAGGTTCAGACAGCTGTGGGGGGACAAGTGTGTCAGAGCTGACGGACCTGCAGGAGTTGAAGGCGAGG
GAAAGTGAGGATGAGGAGGAGAAGGAGGAGAAGGAGAAGGAGGTAGTTACTGCCTCTAAAGGTCTGAG
AGGGGACCATAAGAAAAAAGAGAAAGAGAGTTTGGATCAGGAGAACAGCAAACAGAACAGCAGTGCAG
CATCCAGCTACACAGACCTGTCACAAACCTCATCACCGTCGCTGTCAGAACAGCTGCGTCTGGGCCGAGAG
GACAGCACAGGGTCTGGGATCAGCGTGGAGTGTAAGGTCTGCGGGGACAAGGCCTCTGGCTTCCACTATG
GCGTGCATGCCTGTGAGGGTTGTAAGGGCTTTTTCCGGCGAACCGTACGAATGAAACTGGAATATGATCGCT
GTGAGCGTTCCTGCAAGATTCAGAAGAAGAATCGCAACAAGTGCCAATATTGTCGCTTCCAGAAGTGCCTG
TCTTTGGGAATGTCCCACGATGCGATCCGATATGGACGTATGCCTGAGGCGGAGAGGAAGAAGCTGGTGGC
GGGGCTGCTTGCAGAGGAACTGAACCTCAGCAAACCCGGGGGCTCAGACCTGAAGACCTTGGCCAAACA
AGTCAACACAGCCTACCTGAAGAATCTCAGTATGACCAAGAAGAGGGCCCGCAGCATCCTGACAGGCAAAA
CCAGCAGCACATCGCCGTTTGTGATCTATGATGTGGACACACTCTGGAAGGCAGAAAGTGGTTTGGTATGG
AGCCAGTTAGTTCCCGGTGCACCCCTGACCAAGGAGATTGGGGTCCATGTTTTCTACCGCTGCCAATGCACC
ACGGTGGAGACTGTGCGAGAGCTCACTGAGTTTGCCAAGTGCATTCCAGGGTTTGTGGACCTCTTCCTTAAT
GACCAGGTGACTTTGCTGAAATATGGCGTGCATGAGGCTATTTTTGCCATGTTGCCCTCTCTCATGAACAAAG
ATGGGCTGTTGGTTGCCAATGGTAAAGGCTTTGTGACCAGGGAGTTCCTGCGCAGCTTAAGAAAACCCTTC
AGTGAGATCATGGAGCCCAAGTTTGAGTTTGCTGTCAAGTTTAACGCTCTGGAGCTCGACGACAGTGACCT
GGCCTTGTTTGTTGCTGCCATTATTCTCTGTGGAGATCGTCCCGGGCTAATGAACGTGAAGCAGGTGGAGCA
GAGTCAGGACAGCATCCTTCAAGCTCTGGACCTCCATCTTCAAGCAAACCACTCTGACTCAGTCTACCTCTTC
CCCAAACTGCTGCAGAAGATGGCCGACCTCCGTCAGCTGGTTACTGAGAACGTTCACCTTGTCCAAAAGAT
CAAAAAGACTGAGTCTGAGACCTCGCTCCACCCTCTACTACAGGAGATCTACAAAGACATGTATTAGTATTCT
CCAGCACAGACTGTTTTAGCAATACTGTTACAGAATGAAATTTACATTTATAATAATACACTGCATACAGTACA
AGCACTGTTCCACGTACGGGCATGGATTTTAACGTGCAGATGAGGCAAGGTCACGTGGTAGGGCTAACACA
ATCAGCAAAGTCTTCTCAATGTCAGCATTACCCTCAGCACATGTGTGAGAGTGTGTGAGAGTGTGTGAGAGT
GTGTGCACGTGAGTGTGTGTGTGTGTGTGAGAGATGAGCATCTGGAATCATTTGCTGCAAGTTCTAGTTTCC
CACAGCGGTCACTTAACAGAGAGAATGATATGTTTTCTAACCGAGGCTGCTCCGATTACATTCAAATAGCAA
AACTGACCCCAGAGCAGCGTCTAGAGAGAGCTTCTCCTCATGTGTGAAGGGAAAGTATTTCATGTTCTTACA
CCAATGGCATAGTCGAGGTAGAAAAGAGCCGTAAAGTTTGCACTGTGCAAGAGTTTCTTATAACGCCAATGT
CTTTGGTCAAGTGATTTGTTCTCAGACTTTTGGTAAAGCTGAAGATAAAAATTGGTTTTTGTTTACATCGTCAT
CATCATCATCATCATCATCATCACCATCGTCATCATCAGCGACTGCTACTTTGCCATTTGGTTTATAGGACACAA
ACTGGGCTGGCTCCAGCAGTGTAGAGCTCTCTCAAAAGCAGCTGTCTGTGCAATACCGTTTCCATTTGTCAAT
AAACGTGGACATCCAAGATATATAGATTTTGTCTTAAAGCCATCTACCTAAGTGGTAACTAATGGACTATGCTG
CACAGATTTTGTTTCTGAAGCTGACACTATAAACACATGACCCATTGAAGTACAAGGGACCGCATGTTTTTAT
TGTTTTTCTTTAGTTTGACATACAAAATGATCATGAACTTGCCATTTTAAGGGTTTCTTAGTTTATTCTTTGTAA
TTATGTTACTAATTATTTGTAATTTTTGATCATATTTGTAAGGGCAAAACATGAATTATCATCTTGTTTTATGTAC
CAAGTATGTGTTCTTTCATATTTCATTTTACTCTCCTGCCTATTTTAGTAGAGGTTGTACCAAATAGATCATCAA

GCATACCAACAACAAACTTTTCCTTGATGAGGAATTTGGATTTCAAACGCCTTAGCAGGATTTACCTAAAAAC
CAGCATATTGTATCATGATGTCATCATTGGGATTAGAAATTAACATCCATCATCTGGTGGTATGGGTCGTTTATG
TGTTCACCTGAGGTATAACCACAGTCCTAGATTACTATCCTTTTTGTGTACAATCTGATTTGTTTCTTTAGTCAG
GATTCACACCTTTCCAACTGTTTCCCCCTCCATACTCTGTAGGACCTCTTTTGTTTTCACTGTTCTTCTGAATGG
ACTTCAGTTTGTCAGCACTGGTGCACACAGTCCACCTTCAGAAGTCTCCTCTTGTGCTCTTACTCTGTCTTGA
TCCCTATCTACCTCTAATGTATGATCTGTGACATTATTCCACTGTCTCATCTAATCTCTGACAATATTTTTTTTTTT
TTGCCTTAATTTTCTGCTATCGGTACACGTAGCAAAAAATTGTTATTATTATTAGAGGCTGATGTGCAGTGTGC
CAAGATAGCAAAAGGGTGTGTGTGGATGGATAAAACAAGAAAGTACACCAAAAAAAAAAAAGTTGATGTA
AGCTACCTCTATGCCTGTGTAAGTGATGGATTGACTTGAATGAATTTCGTCCTTATAGTCCCATGTAACCTATCT
GCAGATCTAGTTTAGATACATCTCACTTATAAAAAATTATTAGAAATATc

PLRP

gACTACCATGTTGTCTGGCCTTGAGATGTCTCAGCTGTACAGTTTGGGTCTGCTCTGCTTCTTCATTGG
CGCATCATGTGCGGCCGAGGTCTGCTTTAATGATCTGGGCTGTTTTGATGACCTGCCTCCCTGGGGGG
GCACTGCTCAGAGACCAGCCTCCATCCTCCCCTGGCATCAGGAGGAGATTGGGACTCGCTTCCTCCTT
TTCACACAGAAGAACCGCTACTACCAGGAGATCAAGGCTGATAACAGCATCCAAGCATCAAACTAC
AGCGGGATTAAAAAGACTCGATTCATTATTCCTGGTTATTTGGAAAAAGGAAATGAAGATTGGCCAC
AGAAGATGTGTAAGGTTATGGTGTCGTGGGAGAATGTGAACTGCATCGCTGTGGAGTGGAAGAAAG
GTGTGAGGACTCAGTATGCCCAGGCTGCCAATAATGGCAGGGTGGTGGCTGCCCAGATGGCATCCAT
GATCAAATTCCTCATGGGCAACTATAAGCAAACGGCTGATAAGTTCCATATCATCGGACACAGCCTT
GGAGCTCATGTTGCAGGAGACACTGGCAGCAGAATCCCTGGCCTTGCACGCATCACAGGACTGGATC
CAGCTGAACCTTACTTCCAGGACACTGACCCCTCTGTGCGTCTGGACACCAGTGACGCCGTCTTTGTG
GATGTTATTCACACCGATGGACATCCTTTCAACTCCAGACTTGGTATGGGGATGTCACAATCTGTGGG
CCACATTGACTTCTACCCCAACGGAGGAGAGCTGATGCCTGGCTGCTCCGCTAACAAAGGCAAGCCC
ACTGACCTGGACGGCATCTGGGAGGGTACCATAAAGTTTGATGCCTGCAACCACGTCCGAGCACACC
ACTACTACAGTGAAAGCATCGTCAAACCCCAAGGCTTTGTAGGATATCCCTGCTCTGATAAGGACAG
TTTTGCTGCCGGAAAGTGTTTCCCATGTGCAGACAACAAATGTCCTCTGATGGGCCACCAGTTGACTG
TGACTGATGGCATTTTGAAGACCAAGTACTTCCTCAACACAGGCAGTTCAAATCCCTTTGGCCGTTAC
AGCTACAAAGTTACAGTTACCCTGGATGGTCCCAGCTGGCCCAATCCAGGCTTCATGTATGTGGCAC
TCACAGGAGACAATGACAGCACCAAAGAGTACCAGCTTCATGTGGGTACAATGATGCCTGGAAGGA
ACTACAAGTTGCTGATTGATGCTGAGGTGGATGTTGGTGATGTGACAGCAGTGAACTTTCGATGGAA
CAACTATATCTTTAACCCCTTGAATCCCAAGTATGGTGCATCCAAAGTGGAGGTGCAGAGAGGAAAA
GACAAGAAGATCATGTTCTTCTGTGGAACACAGAACGTGGTAGAGAATAAGATCCAGTCTGTACTTC
CATGCCAAGCTTAACCAGCCAGAATCACCATGTCACTCAGGGTCTCTCAATTAAAAAAAATGCATTC
AAACCgc

SCD1

gGTCCACTCTCCGGGTTTACTGTAGTTTTGCGGGGTTAAGCAGACCAGCTCCATCTCTGTGTTAACAA
GCCAACTCTCGTTTTCTACTCGTCTGCACGTCTTTGCTCAGAGCATCACTAAACCGCTGCCACGATGA
CGGAGACGGAGGCGTTGGAGAGGAAGCAGCAGCAACACAAGCCCAGCAACGTGCATGTTATCCCAG
AGGCCAACAGAGAAGACGTGTTTGATCACACATACAAAGAGAAAGCGGGGCCCAAACCTCCCATGG
TCATCGTATGGAAAAATGTTATATTGATGTCGCTGTTGCATATAGGTGCCCTGTACGGCCTGACCCTT
ATCCCCTCCGCATCTCCTTTGACCTTGCTTTGGGGATTATTATGTTTTGTGATAAGTGCTTTAGGAGTT
ACTGCAGGAGCTCATCGCCTGTGGAGTCACAGATCCTACAAGGCCTCATTACCTCTAAGGATCTTTCT
TTGCTTAGCTAACTCCATGGCATTTCAGAATGATATCTTTGAGTGGTCTCGAGACCACAGGGTTCACC
ACAGATATTCAGAAACAGATGCTGACCCTCACAACGCCGTGCGGGGCTTCTTCTTCTCTCATGTCGGC
TGGCTGCTCGTGCGCAAACACCCCGACGTTATTGAGAAAGGACGCAAGCTGGAGCTCAGTGACCTGC
GCGCTGACAAAGTTGTAATGCTTCAAAGAAAGCATTACAAGCTCTCCGTGCTAGTCATGTGCTTCTTA
TTCCCCATGTGGGTGCCTTGGTACATGTGGGGGGAGACTCTGTGGGTGTCGTACTTCATCCCAGCTGT
GTTGAGGTACACCATGATTCTGAACGCCACCTGGCTGGTCAACAGCGCGGCTCATATGTGGGGAAAC
AGGCCCTATAACAAGAACATCAGCCCCACGGAAAACATGTTTGTCACGTTCAGTGCAATAGGTGAGG
GATTCCACAATTATCACCACTGCTTCCCCTATGACTATGCAACCAGCGAGTTTGGCTGCAAGTTGAAC
CTTACCACTTGTTTCATTGACTTCATGTGCTTTTTGGGCCTGGCAAAGGACCGCAAGAGAGTGCCCCG
TGAGGTCATCATGGCCCGAATACAGCGCACCGGAGACGGAAGCCACCGGAGTGGCTAAGATTGTTG
GATTTTGGTCGGCTTCAACGCAAAACAATCAAAAAGAAGAAGCTTCACAGTCTTTGACAGCTATACA
TTTCCCATCCCCTGAGGATAAAAGTCAGTTTTCATGAGGGCTTGTTGCTTGTTTTTTTGTGGTTTTTGT
AGCTGTAACTAAACAAAAAAAAGTTTTAAGATGGATAACGAACACATTTTGCTAAGCTCTTGGCCTT
GGTTTCTATTATCGAGGCCTAGGTCTTGCAAATGTGTTTTATATTTGAAGTAGATCCATTTAAAAAGT
CAACTGTTTTGCCACTTTGGTGTGCATTCTTCATGCCAAGTCATGTAGCCATCATTTTTATGTAAGTAT
TACTCAATTATTATTCGAAATAGACAAGAAATGGAGCACTGAAATATGGAGTAACATTTCATCGAAT
CTGGTGATATGGTTGCCTTTTATTGCAGTTTAACAGCAAGCTTCTAGAGTTCTGATATAAATTCACTTT
CTGAACGCTCTAAGATCTGAAGAAAAGGGAATGAGAATTATCCTTTGCTAAAAATGTATGAAATATG
GCACATGATGAAAGAGTGAGGGAACCCAGACCATCTTATAACCTGACTATACCGGTACCCCAAAACT
GTATGGAAGTCGAGTCATGCAGTGTGTGTTGCAGTGTGTAACTTGGTTTTCTTTTAACCAAACCAAAT
ACGTAGAAAAGTCAAAGGCTTTAGGGAAATACACACTGTATTTAGTTTGTGTTATAATCATAGTTTTC
TCCAGACAATATGAGGGACACATTTATCATGTAATTTTAAACTGCCATTCAATATTAACAGAAAATG
ATAAATGGAATTTTAACATAGATCCATGCAATATTTAATTGACTTACTTGTGTTTTTAATGAATATAT
CATAGTGCCAAGAGCGATCCAATGGTCGACATATTTATAACCATCTCAATGGTCCACTAGAGCACAC
GGTCAATATTCTTCACTCAAAATCATTGTGGAAGTTTTTTGTGGCATCAGTGGCTTTGCTTCCTCTTGA
GATTTTATCCAACATGTTCTTCTTCTGTTCTTCTGTCCTGAGACAACTCCAGCAAGAGATCTTGCTGGT
TGGACGTATAGCAGCCAGTCATGTGACAGTTCTGGTGTTACAAAACAACATGCCACCCATGCATGAT
GATATACTGTAGTAGGTGTTGTAGTGAGGTGTTTCTCCGGTGACTCTAATGGCAGAGCTGTGTCTATT
TTTGTACTGTACTGTGGAGCTGCATCGCTTTTGGATCATCAGGACTGTTGCCGTGGTGATTTCTGAGA
CGGGGGTCTGGTTTCCCTTTGATGTCCTGCAAAAAAACTGATTGTCATAGCAAAAATTTCAGGTGCAT
AGATGTTTATGTTCATCTTCAATTCTGGTGTGGGGAATGCTTATATTATTTATGTATTTTTTTCTTTTTC
TTTATTGACTTAAAAAAAAAAAGATTTCTCTAATCGGAACCGTTTCTTCTGCATTCCAACAATTGGCA
GACAGTCTCGTAAAGAAAAATATATTTATATCAGATTGGAGCAGTCGTTTATTTCTTTCCCAAGAGTC
AGGATACAACCTAATGCCTTATGACTTGCATCCATTCCTGATCGTTCTTCACGTGCTGTTGAAACTGA
GACAAAAAGGTTCAAGTACCACAAAACAAATCTGCCAGAGGTCCATGTTTACAGGCCGAAGCACCA
GACAGAACACTGTTTTTGAGCTTTTAATTTAGGCAAACGGTAGCATGACAACATTACAGTAAATGTA

AACATTACCTCTACTGTATTTGCAGTGCTGTAAATGCAAATAGATGATAGAGGTTAGTTTTCAAAGA
ATCAGATACAAACGTGACACCTGGTGCAAAATATTTGCCCTGATGGATGTGCCAAGTAGTTTCAACC
CATCATGGCATCTCAGTATGAAAACTTCCAAACAAAAGGTGCTGTCATGAGGAATCCTTATAGCACC
TAACACCTGACAAGATATCACAAAGCCATGCAAACAAGTCCTGCTGCCTGGAGCGCCAAAACCTTCC
TAATGATGAGGGGGATGAAGAGGTTGTCACTCAAAATGATTATGTCAAATCTCAACTATATTGTATT
CTGAAATAAAATTCACAATATTTTCAATACACAATAATTC

CPT1a

gAGTCTCCGCCGACAACCAGGCCGTGTCACATCCTCGCCAGAACAGACAGCTCTGCTCTCTCAGACTG
ATAGAAAGGGTTGTGACCCTATAAAGTAGCCACCATGGCAGAAGCGCACCAGGCGGTGGCCTTCCA
GTTCACCGTCACCCCCGATGGCATCGACCTCCAGCTGTGCCACGAGGCTCTACGCCAGATCTACCTCT
CTGGCCTCCACTCTTGGAAAAAGAGGTTCATTCGCTTCAAGAATGGGGTAATGACTGGTGTGTACCC
AGGCAGCCCTGCTGGGTTCATGGTAGTGGTTGTGTCCTACATGTCCTACAATAAATATCAAAAGCTG
GATCCCTCTCTGGGGTTGATAGCCAAGCTGGGCCAACACATACCCATCAGTCGATATATGTCCACAG
ACAGCCAGAAGATAGTTGGAGGAGTTCTGGTGGGCACGGGCCTGTGGGTCACTATAATTATGATTAT
GAGGAGTGTCCTAAAGGCCCTGTTGTCGTGGCATGGCTGGATGCAAGCACGCCATGGCTCTGTGTCC
TGGTCTACTCGTGTGTGGATGATATTAGTGAAGGTGTTCTCTGGCAGGAAGCCAATGCTGTACAGTTT
CCAGAACTCCCTGCCACGGCTACCTGTTCCCTCTGTCAAGGACACCTGCAGAAGGTACCTAGAGTCA
GTGCGCCCACTGATGGATGATGAGCAGTATGAACGGATGAATGGCTTGACTAAAGACTTTGAGAAG
AACCTGGGACCCAGACTGCAGTGGTACCTCAAACTCAAGTCCTGGTGGGCTTCCAACTACGTCAGTG
ACTGGTGGGAGGAGTACATTTACCTCAGAGGCCGTGGACCCATCATGGTCAACAGCAACTACTACGC
CATGGATTTCCTGTATGTGTTCCCCACCTCCATCCAAGCCGCCAGGGCAGGTAATGCCATCCACGCTA
TCATGCTCTACAGGAGGAAACTGGACAGAGCTCAGATCAAACCACTCATGCTCCTACACACTATTCC
CATGTGCTCAGCCCAATATGAGCATATGTTCAACACCAGTCGTGTCCCAGGTGTAGAAACAGACACC
CTCCAGCACATGAACGAGAGCAAACACATAGCTGTGTACCATAAGGGCCGTTTCTACAAGGTGTGGA
TGTTTTATGATGGGCGGCTGCTGTTGCCACGGGAGATAGAGCAGCAGATGGAGAGGATCTTGGCAGA
CGAGTCAGAGCCCATGCCAGGAGAAGAGAGACTAGCTGCACTTACTGCAGGGGAAAGGACCCCGTG
GGCCAGGGCTCGTGAAACCTACTTCAGCCGTGGTAAGAATAAGCAATCTCTGGACGCCATTGAGAAG
GCAGCCTTCTTTGTTACCCTTGATGACACTGAGCAGGGCTACGACGAGGCAAACCCAGTCAAGTCTC
TGGACAGTTATGCCAAGTCCCTGCTCCACGGAAAGTGCTATGACAGGTGGTTTGATAAATCCTTTAA
CCTGATAGTTTTCAAGAATGGTACCATGGGACTCAACGCAGAGCACTCCTGGGCAGATGCTCCGATC
GTTGGCCATCTGTGGGAGCATGTACTTTCCATGGACCCTGTTAGGCTGGGATATACTGAGGAAGGTC
ACTGCTGTGGACAGCCCCACCCCAACCTGCCAGGTCCTCAGAGGCTGCAGTGGGACATCCCTACTGA
GTGCCAGGATGTCATCCAGAGCTCTCTGGCATTGGCCAGGACTCTGGCTGATGATGTGGACTCTCAC
ATTTTCCCCTTCATTGACTTTGGAAAGGGTCTGATTAAGAAGTGTCGCACCAGTCCTGATGCCTTCAT
CCAGATCGCCCTACAGCTGGCACATTACAGGGACAAAGGGAAGTTCTGCCTGACATATGAAGCCTCC
ATGACACGTTTGTTCCGTGAGGGCCGAACAGAAACTGTGCGCTCCTGCACCGTGGAGAGTTGTGCCT
TCGTCCGTTCCATGATCAGAGATGAGACAACAGAAGAGCGTCTCAAGTTGCTCAAAGTGGCAGCAGA
GAAGCACCAAAACATGTACCGCCTGGCAATGACAGGAGAAGGCATCGATCGCCACCTTTTCTGTCTT
TACGTGGTTTCCAAATACCTGGGAGAAGACTCAGCCTTCCTCAAGGAAGTACTGTCTGAGCCGTGGA
GATTGTCCACAAGTCAGACCCCTCTGCAGCAGACTGAGCTGTTTGATCTGGTCAAACACCCTGAGTA
TGTGTCCTCTGGAGGTGGATTTGGTCCTGTGGCTGACGATGGTTACGGTGTCTCCTACATCATTCTTG
GTGAGAACCTCATCAACTTCCACATCTCCAGCAAACACTCAAGCCCAGAAACCGACTCCCACCGTTT
TGGCACCAACATCAGACAGGCCATGCTGGACATGCTGGCTCTCTTCCAGCTTGACAAGAAAGCCAAG
TGATTGTCCTCTCCATAGGTGAAATAAACATGAGAAGTTGTTTTTAAAAAAAAAAACATATATTCTTC
CTGAAGTTATACAGAATCAAATGTTGGATTTGGGGTTATTTTTTAAGTTAATTGATGTTTCAGAAATA
ATGATGATTGAAAGGAAATATTAATGTCAAGATGTGTGTGAGGCATTTATGTGTACTGTATGTATAG
GCATGGTGAGATTCAGATGGAATTGGGTACAGGTAGTGCACAGATCAAAGGTTTTATTTACAGGACT
GTACACACCGGTGCCTTAGTGACCCAATAATGTAAAAAGGGCAGAAAGGGAGGAGAAAATAACAGC
TGCTGTCATCTGAGATCAAGACTGTATTACCACAGTAAGAGACATTTGAGTTGAAGGATTGCTCAGT
ACCATCATCCAATATTTGTATCATGTTTTAAGTTCAGAGATGGTGCAATATTTTTACCAGTTTTTGTCC

CATTGTTGAAAATCCTTTGAGTCCTTTTTGTTTTTAGGTTTCTTCTGGTTTCCTCAATTGCAGAACATA
AGTCATGTCTAACTGGCCAAAGCAGCAAATTGTGTGTTGCATGGTGTCCATTAATTTCATATGGTATT
GTTATTTTTTTATTTTGCCAGAGTCTGATTTGGTTGACTGAACAGTTGGGTCCAGTAATTTAAACCTAA
TTTTTGTATTTTTAATCTAAGACTCTAGGAGTAAAACATCTGTAATTACAAGAATGCTAAATATTAAT
CTTGCGTGACTGTGTGTCAGTGTGATTCTGGATACCAACAAAGACAGACAAGCTGTTGCCACCGTCT
GCATGAATGCATCTTCATTGTTTTTAAGCACACAGTCAGTATGTTTGTTCTTTTTAGCTGCCATGATTT
TACGATGTAGTGATGAACACAGATCATGTTTGTGTGTGTTTGTCATTTTCATACTCAGGTTTTATCAA
CTTGGTACTTGAGGGCGCTTGCTGCAGCAAGATAAGTGCTTATGCTGGCATGTGCATGTGCAACAGT
AGAGTCAGTACAATGTCATGTGACCCAAATGAGCTGGGGGGTAGTATAACATGAAGTTTGCTGTGGA
GGTAAGTGACTGGCAGGACACGGTGTGTGTCTGGGTTGGATCTCACTTGCACATGTTGAGTTTGATTG
ACAGTGTGTGGTCGACAGTGACTTTTTTTAGTTTCTTGTGTTGTTAAAAAGAGTGGGTTAACTGTACT
ATAACTCTGTTCTGCACACCATGACTGGCCCTTCAGTTCAAACTAACCAGTTCCAAAATGCCTTCAGT
CAGATCAGGATGGATTTTAAAGTTTTGTGAGAAATTATCAAGATGAAGTGCAGAATTGCTTAATAGG
ATGTTATGTCCATCCACTACAGTAGGAAGTCATTTTCAGATATAGAGGTGCAGCTGTCATGTTTAGGG
ATGCATTTTCTGTTATTATGTATATGTTGTTAGCTCATCATATTTATTTTTTACACGTTTTCACCTCTTT
ATTCACAAAACAACAATTTACCAGTTGAATGTGGCAAATGTTAAGCATTAAGTTAAAGCTTTGCTGA
TTCAAGAAGTGAAACTGACAAAATGCTTCCTTTTCTTTCTTTTTTTATTTTATTTTATTATTATTATTTT
TGGACATGACATTCATTTTGTCATCAGACTCTGCCAGTATGTTGTAGTTGTATGACTTTTTATCTCAGT
AGACCTCATGTTTACACTGCTGCGTTGTGTTTGAAGATGAAGCTGTATATCTTTGCCTTTATTTAAAG
ATGTGTTAAAAGACAAGCAGCTGATATACCAAAACCAGTTTTAACAATAAAGTGAATGTCAGACAGC
TGTATCg

PPAR α

gAGTGTTGCGGAGAGCACGTTACACGTCCGGACTGAGCCGTGGCGTCCCGAGCCACCGGGACGTGAA
CGTGTCCCTGTGTGCGGGGGGAGGTTCCCGCACGTCCATTGGTTGAAACCATAGGTGTGTCCTGGGG
GGGGGTGCGTGTGTGTGCGTGCGTGTGTGTGTGTGTGTGTGTGTGTGTGTGTGTGTGTGTGCGCCA
CGGTCGCCGTGCGTCGCTACGTTACTTCCTGTTAGAAGATGAACGGCGGTTTGACAGCACGCAGGGA
GGCGACGGTTTTGTTTTGGTTTTTTATAAGTTGGATCCCGGGACCAGAACACCTGCGTCCGCTGACAG
ACAGCCGTCCATCCCGGTGACAGCATGTGGAAATGTTCCGGATAACAGCTTCTGAGTCTGGTGAGTG
AGGGGTTGTTCTTCCAGGTTTTGTTTGATGTCCACCATGGCAGTGGATCTCTTTAGCCCGCCATCCCC
ACTGGGGGATTCTCTGCTGGATAGTCCGCTGTGTGGAGACCTGATGGAGGATCTACGTGACATCTCT
CAGTCCATAGAAGACGACACGCTGGGATTTGATTTCCCAGAGTACCAGAGCACTGGATCGGGGTCCG
AGAGCTCCATCACACTGGACACGTTGACCCCGGCCTCCAGTCCGTTGTCCGGGGTGTGTGGAACAGC
ACCAGGACCAGAAGAGAGCTTCACCCCCCTCAACCTGGAGTGCAGGGTGTGCTCAGACAAGGCTTCA
GGCTTCCACTATGGCGTGCATGCCTGCGAGGGCTGCAAGGGTTTCTTCAGGAGGACCATCAGGCTAA
AATTGGAGTATGACAAGTGTGAACGCAACTGCAAAATCCAGAAGAAGAACCGCAACAAGTGCCAGT
ATTGTCGATTCCACAAGTGCCTCTCTGTGGGAATGTCCCACAATGCCATCCGGTTCGGTCGGATGCCT
CAGGCGGAGAAGCTAAAGCTCAAAGCAGAAAGCAAGATGGTGGAGAAAGAGGTGGCTAGCCCCAT
GCTGGCTGACCACAAGATTCTGGTAAGGCAGATCCACGAAGCCTATATGAAGAACTTCAGCATGAAC
AAGGCGAAAGCTCGGCTCATACTCACTGGAAAGACCAACAAGCCGCAGCCATTCATCATTCACGACA
TGGAAACGTTCCAGCTGGCAGAGAGGACATTAGCGGCCCATATGGTAAACGGTGACCATCCGGAGC
CTGACAGCGGCCTCCAAGCTGGGAAAGTGGTTCCTGGTGTGGGTTGTGGGGAGCTACAGCAGAGGG
AGGCTGAAGCCAGGCTCTTCCACTGCTGCCAGAGTACTTCTGTGGAGACGGTAACAGAGCTGACGGA
GTTTGCCAAGGCGGTGCCAGGTTTCCAGAGCCTGGATCTGAATGATCAGGTGACTCTCTTGAAGTAC
GGTGTTTACGAAGCCCTCTTCACCCTCCTGGCATCCTGCATGAACAAAGACGGCCTCCTGGTGGCCCG
TGGTGGAGGCTTCATCACCCGAGAGTTCCTCAAGAGCCTCCGGCGGCCATTTAGTGACATGATGGAG
CCCAAATTCCAGTTTGCCACACGCTTCAACTCCCTGGAGCTGGATGACAGTGACCTGGCCCTTTTTGT
GGCTGCCATCATCTGCTGCGGAGATCGCCCAGGCCTGGTTGACGTGCCTCTGGTGGAGCAACTGCAG
GAAAGCATCGTTCAGGCACTACGGCTCCACCTGCTGGCCAACCACCCCGACGACCACTTCCTCTTCCC
CAGACTGCTGCAGAAACTGGCTGACCTCCGGGAGCTGGTCACGGAGCATGCTCAGTTGGTGCAGGAA
ATCAAGACAACAGAGGACACGTCGCTGCACCCGCTCCTGCAAGAGATATACAGGGACATGTACTGA
GAAGACAGCACGTAGACTCGTGAACATAAATATTAACCGATACTAATCGTGCATATCAGTGTGCTGT
AGCTTATTGTTCTGTGGATACACTCTGCTGAGTTTGAATTGAGACCATGTGGTTTCCTACTGGAACAA
AACACAAGTGAAATAGGGGAAAGGCAGCTCTTACAGATGGCGTGACTGCTTCCATGAAAGGGGGGC
ACAGTGTGGAAGAGACTTTTTGATCTTACACCATAGATGTGTAGAGATATTTGTTATTTGCTGAATTT
AGGACATCCAATTTTGATTGATCAGAGTCCTGGATGCCAGATTTTCCTCTGCAGACACAAGAACTGA
ACAAAAGCTAAGTAGGGAGTGGGCATTAAAGATGGGCATACACATATTAAAGATACCAGGACATGA
CTGTTGAAGTCGTGCATGCATTGATGCTAAATATTAATTTTGCTTGTGAAGAAGCAAGAGGCAGAGA
AGTTAGAGAAGTTATAACATTTTTAATCTTTGATAACTGCAGTGTTGTCAATACCAGACATATTCCTA
ATATTTTGGATATTTAGTGCGTATGATATGTTAATTTAAATTTTGGATTTGGTTAATATCATTGCCCTT
TTTATTGTATACTAGTGCCTTATTGATCTCTCTTGACTGTTTTTAGATTAATGGTTAAAGAAAAACTAC
ATCAGTATTCACCAAGTGCCATAATGCCTCAGTGCCATAGCTCATATTTCCTCTTTGGACCATACCTT
ATAACCCCTGGGTAGATCTGTAACTGTTTGCAGCTGGACCAGGAAATGTTCAATATATTTGTTCACAA
TATTACATCTTACAATATGGCTGCTTATATCTCACAATATAAGCAACTATCATTTGAAACTGTTTTAA
TATGAGATGTGTATTTATTTTAATCCTGTATCCTACTGATAAAAACCAACACAACATGATAGCTCACC
TAACCTTTTTTTCAATGTTACAAAGGACAAAATGTTTGGTACAAATCCTGCCATCAGACTCATTGTTT

TCAAGAAAAAAAAAAAAAAAAAAAAAACTGCATTGGCCAGTACTGAATATTTTGACCACAAATACAA
TAATCTGTTAAGCTGGTTTATGTGTTATTTTGTAAATATAACAAGAATGTGGTGGCCGCAGTGAACTT
TCACTGTTGTTTCTTGTCATAACAGACGGTTTATATTTTTTAATCATGTACGATGCGGTGTACATTAAA
AAATACATTTGCAGTTTCTATAAAAATAATTGTCACGTGGTAGCCTAAAATAAACTAATATATTTAAC
AG

FAS

gGGACTGCGCGGCCAAGCCAAGCTGTCAGCCCATGTGGCGTGTCCAGGCAGAATGTGAGACATTTAA
ATTCATCAGAGAAAAAGAGTCAAAGGAGCCCGAAGGCTAACGTCAAGCTTATTGACAAGGAAACGA
CAGAGACAGACATTCCTCCCCGTGGATACAGTAAGAGTAACAGCTCCGTGGATCATCACAGGATAGC
GAACGAAAATTAATAGAATAGATGCAAAACAAGCGCTAACATTTCTCCAAGTGGTTCGATTTAGTGA
TTTTTCAGGGACATCCAAAATAGTCTGGACAACACAGCATGGATGAAATAGTTATAGCAGGAATATC
GGGCCGTTTGCCTGAGTCTAACAACCTGGAGGAATTCTGGGAAAACCTCATCAATGGTGTGGACATG
GTGACAGAGGACAACCGGCGATGGACACCAGGTCTGTATGGTCTACCAAAGAGGAATGGTAAACTG
AAAGACATCAGCCACTTCGATGCAGCCTTTTTTGGAGTCCACCCTAAACAGGCCAACACCATGGACC
CCCAGCTCCGTCTCATGCTGGAGATCGCCTATGAGGCTATCATAGATGGAGGGCTGAACCCGGCCAC
GATGCGTGGCAGTAAGACGGGCGTCTACATCGGGGTGAGTGGCTCAGAGGCTGGCGAGGCGTTCAG
CAGAGATCCAGAGGAGCTCCTGGGCTACAGCATGACCGGCTGTCAGCGGGGCATGTTGGCCAATAG
ACTCTCCTACTTCTTTGACTTCAGCGGCCCAAGCACTGCTATCGACACAGCCTGCTCCTCCAGCTTGC
TCGCTTTAGAAAACGCCTTTCATGCCATTCGCCAGGGCCACTGTGATGCTGCCCTGGTGGGGGGAGT
CAACCTGCTACTCAAGCCAAACACCTCAGTGCAGTTCATGAAACTGGGCATGCTCAGTCCTCAGGGG
ACCTGCAAATCCTTTGACTCATCAGGAAATGGATACTGCCGTTCTGAGGCAGCAGTGGCAGTACTGT
TGACCAAGCGGTCGGTGGCTAAAAGGGTCTATGCCACAGTGGTCAATGCTGGAAATAACACAGATG
GATACAAAGAGCAGGGTGTAACATTTCCTTCAGGTGAGATGCAGCAGAGGCTGGTTCAGTCTCTCTA
CCAGGAGGCAAATATATCTCCTGAGCAAGTGGAGTATGTTGAAGCCCACGGCACTGGTACAAAGGTC
GGTGACCCACAGGAAGTGAATGGCATTGTTAATGTGTTCTGTCAATCAAAGCGAGAGCCTCTGCTTA
TTGGCTCCACTAAGTCTAACATGGGTCATCCAGAACCAGCCTCTGGTCTGGCTGCCCTGGCAAAGGT
GTTGCTTTCTTTGGAGAGAGGAGTCTGGGCTCCCAACCTGCACTTCAACAGTCCGAACCCTGATATTC
CTGCCCTCACCGATGGTCGGGTTCAAGTAGTTGACCGGCCTATTCCTGTTCAAGGTGGCATTGTGGGT
ATCAACTCCTTTGGATTTGGAGGTTCAAATGTTCACGTGATCCTTCGTCCAGCCAAGAAATCAGCTCA
TGAAACTGCACTGCCTAGAACAGTCCCCAGGGTGCTTCAGGCCTGTGGCCGCACAGAGGCTGCGGTG
AAGGCCATGCTCCAAAAGGGGAAGGAACACACTGTAGATGACAGCTTCCTGTCTTTGCTGAATGAAG
TGTCAGGGGTTCCAGCTGCCAGCATGCCCTACAGAGGCTACACTCTGATTGACTCTCAAAGTGATGT
CATTGAAGTACAGCAGGTGCAGGCCACTGGCAGGCCACTTTGGTACATCTGTTCAGGTATGGGAACA
CAGTGGGCAGGTATGGGCCGCACTCTCATGCAGCTGCCAGACTTCAGAAAATCCATCTTGCGGTCAG
ATGCAGCCCTGAAGGACACTGGCTTGGTTGTGTCTTCCCTACTCATGGAGGCAGACGACGCCACTTTT
GAAGACACTGTTCATGCCTTTGTTGGCCTTGCTGCTATACAGATAGCTGAGATCGATCTGCTCACTAA
GCTGGGTCTGCAGCCTGATGGCATCGTAGGACACTCAGTGGGTGAGCTGGCCTGTGGCTATGCTGAT
GGCTCCCTCAGCCACAGTGAGGCCATCCTTGCTGCCTATTGGAGAGGCCGCTGCATCAAGGAGGCCA
ATCTTCCTCCAGGAGGCATGGCAGCAGTTGGGTTGACCTGGGAGGAGTGTCTGGCTCAGTGTCCTCA
GGGAGTGGTCCCAGCCTGCCATAATGCTGAAGACACTGTCACCGTCTCCGGTCCTCAGGAAGCAATT
AGTAAATTTGTGCAGGAGCTGAAAGAGCAGGGAGTGTTTGCAAAGGAGGTGCGCAGCGCTGGCGTC
GCTTTCCACTCCTACTACATGGCCTCCATAGCTCCAACCCTGTTGGCTGCTCTGAAGAAGGTGATCAA
GGAGCCACGACAGCGCTCATCCCGCTGGGTGAGCACCAGCATCCCTCAGTCTGAATGGGACTCTCCT
CTGGCTCTGTACAGCTCAGCTGACTATCATGTCAACAATCTGGTGAGCCCTGTGCTGTTCCAGGAGGC
TCTCAGTCTGGTACCTGAAAATGCTGTGGTGGTGGAGATAGCACCTCATGCTCTGTTGCAGGCCATCC
TAAAGCGCAGCTTGAAGCACACTTGTTCCATTCTTCCCTTAATGAAAAGAGGCCACATCAACAACCT
CGAGTTCTTCCTCTCAAACGTTGGCAAAATCTACATGAATGGCATCAATGTGGATATTAACAACCTCT
GCCCAGAAGTGAAGTACCCTGTGCCAGTTGGTACCCCCCTGATCTCCCCCTTGATGCAATGGGACCA
TGCTCAGACCTGGGATGTGCCCAAGGCAGAGGATTTTCTTTCTAGCTCAGGAGCATCCAATTCTGCCA

CTGTCTATAACATTGACATAAATCCAGAATCTACCGACTACTACCTGATTGGACACTGCATTGATGGG
CGTGTCCTCTACCCAGCGACGGGATACCTAGTGCTGGCCTGGCGTACCTTGGTTAGAAATCTAGGGG
TTGTCATGGATGCCACCCCTGTAACCTTTGAAGATGTGACCATCCATAGGGCCACCATCCTGCCAAA
GACCGGCTCTGTTCAGTTGGAGGTACGTCTCATGCCTGCCACCAACAAGTTTGAGGTTTCAGAGAAT
GGAAACCTGGCTGTCAGTGGTAAGGTGAGCATTCTGGAAGATGCAGCCCTCAATTCATTCCACAGTC
AAATAAATCAACAACCTGCCAAAAACAGTGGAGACCCCAATATGAAGCTTACAGCAAATGAGATCT
ACAAAGAGCTCCGACTGCGTGGCTATGACTATGGGAAGACCTTCCAAGGCATCTTAGAGTCCAGCCA
TGCAGGAGACACTGGGACACTTCAGTGGACAGGCAACTGGATAACCTTTTTGGACACCATGCTGCAG
ATGATTGTGGTAGGGCTATCAGGTCGTAGCCTACGTTTGCCAACCAGGATCCGCTCAGTGTGTATTGA
TCCAGCTGCTCATTTAGAGAAGGTCTACACTCACACTAATGGAAAGCAAGCCATTGAAGTCCACCTA
AACCGTTGCCTTGACAACATTGTTGCTGGTGGAGTCCAGATCTGTGGCCTGCATGCCACTGTGGCCCC
TCGTCGGCAACAGCAGCAGAGTCCCCCCACACTGGAGGAGTTTTTGTTTGTTCCCTATGTAGAGACA
GAGTGCCTGAGAGCCAATGGGAAACTGGCAGAGCAGCTGAGGCTTTGCAAAGGTTTAATCTACAGA
CTGCAGCAAAAGTTGGCCCCTCATGGTGTCAAGCTCTCCATCCCTGGGCTACAAGGGGAGTCAGATG
GCCCTCTTCCCACCCCAGAGCCCTCTGAGCCTGGCCTGGTGCGATTGCTTTCTCTGCTCTGTGGCCTG
GAGCTCAACGGAAACCTTCGATCTGAGCTTGAACAGACTGTGGAGAAGGAGAGGGCTTGCCTGCTGC
AAGACAGCCTGTTGCAGGGCCTGCTAGACAACCTGGCTTTCAGGCACTGCCTGGACACCACTACAGA
AAATTGCTTGCCTGGCAAGATCAAAGTTCTAGAGGCTCTGTCCAACGATGGACAACTCTTCTCTCGTG
TGGTAGCCCTCCTCAACATCCAGCCCATGCTGCGTGTGGATTACACTGCCACGGCGACCAACCTAGA
TCTCCTGGCCCCCCACCAGGCCACGCTGGAAGAGCTGGCGGTGACTTCAGCTCAGTGGGACCCCCTG
ACGAACCCAGCACCTGGAGGTGTAGTAGGAGGGGCAGATCTGGTGTTGTGTAACCACGTCTGGGGC
GGTCTAAGGACAAATCCTGGACTGCTGGTGACAAATCTAGCTTCTGGAGCCAAACCAGGGGGGTTTG
TTCTCCTTCACACCCTGCTGAAGGGAGAGACTCTGGGAGAAACAGTGGCCTTCCTCTCCAGCACCGC
TCAGAGTAGTAGCCAGCAAGGACTGCTCACACAGTCTGAGTGGGAGAAGGTGTTCTCTGAGGCCTCC
CTCAGCCTGGTGGCTGTCAAGAAGTCTTTTTATGGCTCTGCCCTGTTTCTTTGTCGCTGTCGGTCTCCA
GCCAAACAGCCCATTTTCCTGCCTGTGGACAGCACTGACTACAATTGGGTGGAGACACTCAAGGCAA
TGATGGTGGAGTCATCAGACTCTCCAGTGTGGCTGACTGCCTCTCAGGCTCATTGTGGCATTGTGGGA
ATGGTCAACTGTCTACGGCAGGAGCCAGGTGGCAACCGAATACGCTGTGCGTTTGTGTCCAATCTAA
ATGATGCTTCAGCAGCACAGAGTCTCCAGCCTGGCCATAGGTCAATGCAATCAGTACTGGAGGGGGA
TCTGGTTATGAATGTATTCAGAGACGGACAGTGGGGTGCCTTCAGGCACCAGTTCATCACTCACGAT
GTTAATGAAGAACTGACAGAGCACGCTTATGTCAATGTGTTGACTCGAGGTGACCTGTCTTCTCTGCG
ATGGATCGCTTCGCCACTCCGTCACTTTGTGACCAGCAACCCCAACATGCAACTGTGCCGTGTTTACT
ACAGCTCACTCAACTTCAGAGATATAATGCTGGCCACTGGGAAACTACCACCAGATGCTATTCCAGG
TGACCTAGCTCTGCAGCAGTGCATGTTGGGTATGGAGTTTTCTGGTCGTGACCCTAATGGTCGACGTG
TGATGGGGCTGCTGCCTGCTAAAGGCCTAGCAACAAGTGTGGATGCTGACAAACGGTTCCTCTGGGA
TGTTCCCTCCAGCTGGACACTGGAGCAGGCAGCCTCAGTGCCTGTAGTCTACGCCACAGCCTACTACT
CTCTGGTGGTGCGTGGCCGGCTTCGTCCTGGAGAGACTGTTCTTATTCACTCTGGGTCAGGTGGCGTT
GGTCAGGCTGCCATCTCCATAGCACTAAGCAGGAAATGTAAAGTCTTCACCACTGTTGGCTCAAAGG
AGAAGCAGGCCTACCTGCAGGAGAGGTTCCCTCAACTCTCAGCAGAGTCCTTTGCTAACTCTAGAGA
CACCTCTTTTGAACAGCACATCCTGCTCCATACAAACGGCAAAGGTGTGGATGTGGTGCTGAACTCT
CTGGCTGAGGAGAAGCTGCAGGCTAGCATTCGCTGTCTAGCCAGGCATGGGCGATTTCTAGAGATTG
GCAAATATGACCTGTCCAACAATTCTCCACTGGGCATGGCTTTATTCCTGAAGAATGTGGCATTCCAC
GGCATCCTGCTGGATGCGCTCTTTGAAGAGGGCAACCATGAGTGGGAGGAAGTGTACCAGCTGGTGA
AGGAAGGCATTGATGGAGGTGTAGTTCAGCCTCTAAAGACCACAGTGTTTGAGAGGGACCAAGTGG

AGGAAGCGTTCAGATACATGGCTCAGGGCAAGCACATTGGCAAGGTCCTCCTGCAGGTCTGTAATGA
GGAGATGGGTGTAGCTGTCCAAATTGCTTCCCCTTTGTCTTTACCAGCTATGCGTCGCACCTTTTGCC
ATGCCTCACACTCCTACATTATTACTGGTGGACTGGGGGGCTTTGGCCTGGAGCTTGCTCAGTGGCTG
ACAGAGAGAGGAGCCCGAAAACTGGTGCTGACTTCCAGATCAGGGATCAGGAATGGTTACCAGGCA
AAGCGAGTGCGTGAATGGCAGAGTCAAGATGTGGACGTCCTGGTGTCAACTAATGATGTCAGCACAT
TGGAGGGAACAGAGCGACTCATCGCTGAGGCCTGTGCACTGGGCCCAGTGGGTGGTGTCTTCCACCT
AGCCATGGTGTTGAAAGATGGCATGTTGGAGAATCTGACTCCTCAGCTCTTCCTTGATGTTAACAAAC
CTAAATATGATGGCACCATAAACCTTGACAAAGTGACAAGAAAGTTGTGTCCATACCTCAACTACTT
TGTGACATTCTCCTCAGTCAGCTGTGGCCGTGGCAATGCCGGCCAAAGTAATTATGGTTTCGCCAACT
CAGCCATGGAGCGTGTGTGTGAAAAGCGCCACTATGATAGCCTACCTGGACTGGCAATCCAGTGGGG
TGCCATTGGCGATGTGGGCGTGGTCCTGGAGACCATGGGTAGCAATGAAACAGTGGTTGGTGGCACC
CTACCACAGCGCATCACATCTTGCTTGGAGGTGCTCGACCTCTTCCTGTGCCAGCAGAAGCCAGTTAT
GTCTAGCTTTGTACTGGCAGAGAGGACGGTGGTAAAGAGTGAAGCAGGAAGCCAGAGAAACTTGGT
GGATGCTGTAGCACACATTCTAGGTGTGCGGGATGTGAACAGCCTGAATGCTGATGCCTCATTAGCT
GACCTGGGCCTGGACTCCTTGATGGGCGTTGAAGTACTCCAGACTCTGGAGCGTGACTATGACATCA
TCATGGCTATGAGAGAGATCCGCCAGCTCACCATCAACAAGCTGCGGGAGCTGGGCAATAGCAATCC
AGAATCTCACCATGCTACAGCTAAGAGGGATGGCATTCACTCTTTGTTGGAGTCTGATCTGACTCAAC
TATTGGTGAACCCCAATGGCCCCACAGTGATGCCACTCAACAAGGTTCAGAGCCAGGAGAGGCCACT
GTTCCTGATCCATCCCATTGAGGGCTCCACTGTCGCCTTTAAAACACTTGCTGTCAAGCTTAGCATGC
CCTGCTATGGCCTGCAGTGCACAAAAGATGCTCCCCTTGACAGCATCCAGTCCCTAGCAGCCTACTAT
GTGAACTGTATCAGACAGGTTCAGCCAGACGGGCCGTATCGAATTGCTGGATACTCTTTTGGTGCCT
GTGTGGCATTTGAAATGTGCTCTCAGCTTCAAACCCAGAATCTGTCTGTGGAGTATCTCTTTCTTTTTG
ATGGATCACACTCCTATGTGGCAGCATATACACAGACTTACAGGGCCAAGTTGACACCAGGCAAAGA
GTCTGAGGCTGAAACTGAAGCCCTGTGTGCCTTCATCCAGCAGTTCACTGGCATCGAGTACAACAAG
CTCCTAGAGACTCTTCTCCCGCTGTCGGACCTGGAGGCCCGCGCCAACATTGCAGTGGACCTGATCA
CCTCTAGCCACAAGAACATCAGCCGGGATTCATTGCACTTTGCAGCCACCGCCTTCTACTACAAGCTG
AAGGCTGCTGATGGCTATGTGCCTGCAGCAAAATACCATGGCAATGTGATGCTACTTCGTGCCAAAA
CCAGCAGCAACTATGAACAAAACCTGGGGCCTGACTACAAGCTCAGTGAGGTATGTGATGGAAAGG
TGTCAGTCCACATCATTGAAGGAGATCATCGCACCTTCCTGCAAGGAGAAGGGGTGGATTCCATCAG
CAGCATCATCCACAGCTCACTCGCTTAGCCATGTGTTACAGCAAGAGAAGGCTAGACATGACCACAC
CCTCCACTTTCCCACAAATTCCACAACATTCCAAAATGAGTCTCCTCCTCTTATCTGTTCAGGATGTGT
AAGTTTAACTTGCCCTAATAACCCAAATGCACCTTATTCCTCTTTATCGTACATTTTCAAAGCTCATTG
GTCAATTCAACTAATGGCCACTCCCTAACCTAAGCATCAAAGCTCTTAGCTGGTAATGTAATGTGCTG
TCAGTAAGTTTGTTTGTTTGTAGAGTATGAGTAGGGCCTTCTAAATTATGTCTCATTATCTTGCCTTTT
TTTGGATTGCAGTAGGGATGCAAATTTTCAGGATTTTACCTAATCAAGAGTCAGCTCTGTAGCGTTGA
TAACTAATTAATCATTAATCAAATTTTATAGCATATCTCTGTATATCAGGCAAAAGTAGGTATTAGGA
AAAAGCAAAAGTAAAACATTTGAATTATGAATTACATAACACAAAACAATCATTTGAAAAAAATGG
TTGAATTGCTGCTTCAGTCAGTATTCATAAAATGACCAAAGTAATATTCAAGACTCAACTGAGGATA
CAGATTTAACTAATTTAGAACTGCAAATGTCAGAAACAGAACTATAAAATAGACGACCATCATGTAG
CAAAATTTACACCATCTCTGGTGAGTTCTGTGTCGGTTCACTCTAGCTAGCCCAGCTACTAAATCACT
TGTTCAGGTGGAGCCAGTGTCCAGTGGATGTACAAATGCTGTTGAGCCAATTTTTCCTGCCCATGAAA
TCTTTGATTTTCCCTCTGAGAGTGAATGACTGTTACATCCAGAGTACAGTGTCTGTTGTCTTCTGTTTC
AGACCTCACTGTAAGCAGGTGTAGAGTGATTACCTACAAGGAGTAACAGTTTGAACAACAAGATGAT
TCTGATGACTTTTTAGATGTCAAAACACTGCACAGCATTTCATAAAAACTCTGAAAAGTTATTACAGT

TTTGATTTCATCTTTCCCATGCCAGAACAAACACCCAACTCATGGTACAAAATAATCTATTCATTGTT
GGATAACATAAACATTTTTTGTCAGAGCCACTGTGTGTTTTTAACATGTCTACAGTGTGGTGTGTAGT
GTACTTTCCATATCAATACACTTAAAAATGATCTAGTTTAGCATAGCATGAAAAGCTGGCTCTGTAGC
TGTGGTCAGAAAAGTAATTCAAAGTCTGCTTCCTTGTAGCTTTGATCACATTTATGCTGCCAAATGCT
ATTTCATAATTGTACACTGATGCATGTTTACATGTTTGAAGGACTCACATCTTCTACTTCAAATGCTTT
TACTGTACATGTATATGTCCTTACCAGTGAAATCCCACAGTGAAGCTCTGCTTATGTATGGCATCTTT
ACATATGCACCCAAACCTGTTAAAGTCTCATGGTATGATTACTCCTGACCTGCAGTAGTTGGCTAATT
ATTACTGGTAATAGAACCAATTTATGGAAAGCCTCAAAGCTACAAACACTGTCATTAAAAAAATGTT
TTCACAACGTATTACAATCAAGGATAATATTATGAAATGAGCACACTGAATGACTATCCAATAAGCA
ATATGTAATGTCTCATCCACCATATTGAACAACAGTAATAATCTTTATCCTGTAGTACTTCAAATACA
ATTTGTAAAAGGATGTTGCATTTGCTAGACAGATAACTTATTACAGAGCATAACCTGGGTGATAAGG
ATCTGTTCATGAAATAAGATCACATTGACAAACAAGAAAATGTAGATATCTGATTAGCTGAAAAGCT
CAGATCAACTTTGCCCAGCTGATAAACCCATTGATGCCACCACATTGTTGAGTTTATTAGTCTGCTCC
ACAAAACGTTTGTGTGTGAACACAAGGTGTTCTGTGGGTGAAAACTATTCAAAGTTACACTATTTTG
ATAGATCTGTGGTAATGTATTGAAGAGGTTTGGTACATAAATAGTAAATGATGCATCCTCTCCCAAA
TCAGCTTTTAGATTTTGAATGACTACTAAGGCACATATTCTGTTCGTACATAACTGATCTGAACAGCT
GGCCCCTGAGCAGTAAGGCATCTTTGATGAAAAACTGTATTCTCCCAGACCTGTCTCTGACTCCGATG
ACCCTAACTTGGTTTCATGACCCCCAGCTAGCAGTCCAGTCCCACTATTCAACAGACAACCTGACCTC
TGAGACCTCTCATCTGGTCTATGAAGGGTCAGGTGATCAGAAAATGTGGTGATTGATTGCAGTGAAA
AGGAGAAGGAAGTGACAGTATATGTTAACTATTAATATTTCTAATATGTGTTGTGACTAATAAAGGC
ATTTAGCTACTACTgg

ACC1

tgatttTGGTCGCATCCCTCCTGCCGTGGAGAAGGCTATCAAGAAAGAAATGGCTCAGTATGCGAGCAAC
ATCACCTCTGTGCTCTGCCAGTTTCCCAGCCAGCAGATCGCCAACATCCTGGACAGCCATGCTGCTAC
TCTAAACAAGAAGTCAGAGAGAGAAGTCTTCTTTATGAACACGCAAAGCATTGTTCAGCTGGTACAG
AAATATCGCAGTGGCATCCGGGGTCACATGAAGGCCGTGGTGATGGACCTGCTCAGGCAGTACCTGA
AAGTAGAGATCCAGTTTCAGAATGGACACTATGACAAGTGTGTGTTCGCACTACGTGAGGAAAACAA
AGGCGACATGTCCAATGTGCTCAACTATATCTTCTCTCATGCTCAAGTCACCAAGAAGAACCTGCTG
GTTACCATGCTGATTGACCAGCTGTGTGGCCGCGATCCCACACTGACAGACGAACTGATGGCCATAT
TGACTGAACTCACCCAGCTCAGCAAGACAACAAATGCCAAGGTGGCACTGCGTGCTCGGCAGGTGCT
GATCGCTTCTCACCTCCCCTCATACGAGCTACGACACAACCAGGTGGAGTCCATCTTCCTCTCTGCCA
TTGATATGTACGGGCACCAGTTCTGCATTGAGAACCTGCAGAAACTGATCCTTTCAGAAACATCCAT
CTTTGATGTTCTGCCCAACTTCTTCTACCACAGCAATCAGGTAGTCAGGATGGCTGCCCTTGAGGTGT
ACGTTCGCAGAGCATACATTGCGTACGAGCTGAACAGCGTTCAGCATCGGCAGCTGAGGGACAACA
CATGCATAGTAGAGTTCCAGTTCATGCTTCCCACCTCGCACCCAAACAGAGGGAACATCCCCACTCT
AAACAGGATGTCATTCTCATCTAACCTAAACCACTATGGCATGGTGCATGTAGCCAGCGTCAGTGAC
GTTCTGCTTGACACATCTTTTACACCACCTTGTCAGCGCATGGGAGCCATGGTCGCTTTCCGCTCCTTC
CAGGAGTTCACCAGGAACATAACAGACGTGTTGAGTTGCTTCTCTGACTCTCCTCCCCCAAGTCCAAC
CTTCCCAGAAGGAGGTAATCCTGTCCTGTATGGCGAAGAGGACAACAAGAGTGTCCAGGACGAGCC
TATCCATATCTTGAATGTGGCTATAAAGACAGACAGCGACATCGATGATGACGGTCTGGCAGCCAGC
TTTCGAGAGTTCACTCAGTCAAAGAAATCTCTGCTGTTTGAACACGGAATCCGTAGACTGACTTTCCT
TGTGGCTCAGAAGGATTTCAGGAAGCAAGTCAACTGTGAGGTGGACCAAAGGTTTCATAGAGAATTC
CCCAAATTTTTCACATTCCGCGCCAGAGATAAGTTCGAAGAAGACAGGATCTATCGTCATTTGGAGC
CGGCACTTGCTTTCCAGTTGGAACTCAACCGCATGCGCAATTTTGCCCTAACTGCCATCCCGTGTGCC
AACCACAAGATGCACCTGTACCTGGGTGCAGCCCGTGTGGAGGTGGGCACAGAGGTTACGGACTAC
CGTTTCTTTGTGCGAGCCATTATCCGCCACTCTGATCTGGTCACAAAGGAGGCCTCTTTTGAATACCT
TCACAATGAGGCCGAGCGTCTGCTGCTGGAAGCCATGGACGAGCTGGAGGTGGCTTTCAACAACACA
ACTGTACGGACTGACTGTAATCACATCTTCCTCAATTTTGTCCCCACAGTCATTATGGACCCATCAAA
GATCGAGGAGTCCGTGCGCTCCATGGTGATGCGTTACGGCAGCCGTCTGTGGAAGCTGCGTGTCCTG
CAGGCTGAGCTGAAAATTAACATCCGCCTGACTCCAACAGGAAAGCAAATCCCCATCCGCCTCTTCC
TCACAAATGAATCAGGCTACTATCTAGACATCAGCTTGTACAAGGAGGTCACTGATTCCCGAACGGG
ACAGGTGGGGCCCAAAGACCGACAGATCATGTTCCAAGCATATGGAGACAAGCAGGGTCCGTTGCA
TGGCATGCTCATCAACACCCCGTATGTCACCAAGGATCTGCTGCAGTCTAAGCGCTTCCAGGCACAG
TCTCTGGGCACCACCTACGTCTATGACTTTCCAGAAATGTTCAGACAGGCTCTGAAAAAACAGTGGC
ACTCCAGCCAGACCTTTGCCCACTTACCCAAATGCCCTCTCCCTTCTGAGCTGCTCACTTTCACAGAG
CTGGTTCTCGACGCCCAAGGTCAGCTGGTGCAGATGAACCGACTGCCAGGGGGCAACGAGATTGGTA
TGGTGGCATGGCGGATGACCCTGCGCACGCCAGAATATCCAGCGGGACGCGAGATCATCGTCATAA
GTAATGACATCACGCACAAGATTGGCTCGTTTGGGCCCCAGGAGGACGTGCTGTTCCTGCGAGCCTC
AGAGATGGCCCGCGAGAGCGGCATCCCGCGAGTCTACATCGCAGCCAACAGCGGCGCCCGCATCGG
GCTGGCGGAGGAAATCAGACACATGTTCCACGTGGCCTGGCAAGATCCAGCTGACCCCTATAAGGGT
TTCAAGTATCTCTACCTCACACCTCAGGATTACAAGAAAGTTTCAGCCCTGAACTCCGTGCATTGCGA
ACATGTGGAAGATGAGGGAGAATCCAGGTACAAGATCACTGACATCATAGGAAAAGACGAAGGGCT
GGGTGTGGAGAATCTGAAAGGGTCTGGAATGATTGCTGGAGAATCTTCTCTGGCCTACGAGAAGATC
ATCACCATGAACCTGGTCACATGCAGAGCCATAGGAATCGGGGCCTATTTGGTGAGGCTTGGACAGA
GAACCATTCAAGTGGACAACTCTCATATTATTCTTACTGGAGCTGGAGCACTCAACAAGGTGCTAGG

TAGAGAAGTGTACACATCGAACAACCAGCTTGGTGGAATCCAAATCATGCACAACAACGGTGTGAC
CCACTGCACCGTTTGTGATGACTTTGAGGGAGTCTTCACACTTCTACAGTGGCTGTCCTACATGCCCA
TGTGCACATCTAGTCCGGTGCCTATCATCAATTCCAAGGATTCCATAGATCGGCTAGTGGAGTTTGTG
CCTACAAAGGCTCCCTACGACCCTCGCTGGATGTTAGCAGGGCGTCCCAGCCAGACTCCAAAGGGTT
CCTGGCAGACTGGCTTCTTTGACCATGGTTCCTTCATGGAAATCATGCAGCCTTGGGCTCAGAGTGTG
GTGGTAGGCAGAGCCAGACTTGGCGGAATACCTACTGGAGTGGTTGCTGTGGAAACCAGGTCAGTG
GAGCTCTCCATCCCTGCCGATCCAGCCAATTTGGACTCAGAAGCAAAGATCATCCAGCAAGCAGGAC
AGGTGTGGTTCCCAGATTCTGCTTTCAAAACAGCTCAGGCCATTAAGGACCTGAACAGGGAGGGCCT
ACCTCTCATAGTGTTTGCCAACTGGAGGGGCTTTTCTGGAGGAATGAAAGATATGTACGACCAGGTG
TTGAAGTTTGGGGCCTACATCGTGGACGGGTTGAGGGAGTACAGGCAGCCGGTGCTGGTTTATATCC
CCCCACAGGCTGAGCTGAGGGGAGGATCCTGGGTAGTTATAGACCCAACCATCAACCCTCGTCACAT
GGAGATGTACGCTGACAAGGACAGCCGAGGTGGAGTGTTGGAGCCTGAGGGAACAGTGGAGATCAA
GTTTAGGAGGAAGGACCTGGTGAAGACCATGAGAAGAGTAGATCCTGTCTACATGGGCTTGGCTGA
AAAATTGGGAACCCCAGAGCTGAGTCCCCCTGATCGTAAAGAGCTGGAGACCAAGCTGAAGGAGCG
TGAGGAGTTTCTCCTGCCCATCTACCACCAGGTAGCTGTGCAGTTCGCTGACCTCCACGACACACCAG
GTCGCATGCAAGAGAAGGGTGTAATAACGGACATCCTTGAATGGCAAACGTCCCGTCAGTTCTTTTA
CTGGCGTCTGCGGCGCCTGCTGCTGGAGGAGACGGTGAAAAGAAAGATCCAAGCGGCCAACAGCGA
GCTGACAGACGGTCAGGTGCAGGCAATGCTGCGCCGCTGGTTCGTGGAGGCCGAGGGTGCGGTCAA
GGCCTATTTGTGGGATAACAATGAAGAGGTGGTGGGATGGCTGGAGAGGCAACTAGCTGAAGAAGA
GGGCGCAAGGTCCGTCATCGACGAGAACATCAAGTACATCCGCCGAGATCACATCCTCAAGCAGAT
ACGCAGCCTTGTTCAAGCCAATCCAGAGGTCGCCATGGATTCCATTGTGCACATGACCCAGCACATC
TCGCCCACACAGAGAACCGAGGTGGTGCGTATCCTGTCCACCATGGAGACGTCAGCCTCCTCCTAGT
GGGACCCTGGCCTCTCCTCCCTGCCTGGATGAAGCACTGCTCAGGCCGCGTCCACAGCCAAAAGGAG
CCTGGCTCAGCCACACCTGCTCCTGGCTGAGAACCGGGCCGGAAGCTGAGGCTGGAGGGAGCCAAT
GTGAGGGCCCTTGGCAGCTGCTAGGTTACTGATGAATCACTGGCTATGATTCTCACTAAGTGTTCAGA
TGACTGACATCAATTATAGCATTTTGTAAATGGTCTTACATGTACGCTTGTGTTTAGATAAATCCATG
TATTTCATATTCAGATGCAATTcg

ACO

gaggacagtCAGCTGGCTAGCACTGCGTGCAGCTGTCGAAGAGGAGGGAAGTCGACACAGAGCTCAGAG
TCCGAACAGAAGAAGATGGACCATGAAATCGCTGACCTTCCAAGGGGCCCGCTGGACATCTACAGG
AAAAAAGCCTCTTTCAACTGGAAGGAAATGCTCCACTTCATAGAGGGAGAGGAAGTACTGGTATTCA
AGCAATGTTTGTTTAGGACACTGGAGAGTGATCCTCTGTTTGCCCGCCAGCCTGGTGAAGATATCCCC
ATTGAGAAAATGAGAGAGCTGAACTTCCTCAGGGTGAAGCAGCTCATTCGCTACAACTTCTTGACAC
AAGAAGAGATGATGGCCAACCCCTGGAGGATTGTGGTCCTCAATGATTGTCTGGGCATGTATGACTG
GTCCCTCGCAGCCAAGTTCTCTCTCCACAATGGAATGTTTGGAGGGACTGTTGCTAACACAGGATCA
GCGAGACACAGCAAATATGTTAAAGACACTGAAGATTTGACGGTGTTTGGATGCTTTGCACTGACTG
AGCTGAGCCATGGCAGCAACACCAGAGCCATAAGGACCACAGCAACACATGACCCCTCGACCCAGG
AATTTGTGATCAACTCTCCAGACTTTGAGGCTGCAAAATTCTGGGTGGGGAACCTGGGCAAGACTGC
TACCCATGCTGTGGTGTACGCTCAGCTCTACACATCTGACCATGTGTGCCATGGCTTGCACTCCTTCG
TTGTGCCGGTGAGAGATCCCAAGACCCTTCTGGCTCTGCCCGGTGTGCAGGTTGGAGACATGGGCAA
GAAGCTGGGCCAGAATGGACTGGACAACGGTTTTGCTTTGTTTCATAATGTGAGGATCCCTCAGGAG
AATCTACTGAATAAGACTGGAGATGTTACTCCTGATGGCCACTATGTTACCCCGTTCAAGGACCCTA
AAAAGCGTTTTGGGGCGTCTCTGGGTGCCCTGTCTGGTGGCAGGGTGTCCATCACCAGGATGGGTCT
GGTCAACCTGAAACTGGCTCTGATTGTGGCCATCCGCTTCTCAGCCACTAGGAGACAGTTTGGACCC
AAAGAAGCAGAGGAAATTCCTGTTTTGGAGTACCAGTTACAGCAATGGCGCCTGATCCCGTACCTGG
CAGCAGCATATGCTCTTGAACACTTCACCAAATCCATCTTCATGAACTTTGTTGAGTTCCAGATCGGA
CAGATCATGAAGGACAAGAGTGACAGACAAGAAGAGATGGGCAGAGAGATTCATGCCATAGGCTGC
TCCAGTAAACCACTGGGCTCATGGACTGCTCAGAGGGGTATCCAGGAGTGTAGGGAGGCCTGTGGTG
GACATGGATATCTGGCCATGAACCGGCTAGGTGATCTGCGTAATGACAATGATCCAAACTGCACATA
TGAAGGAGACAACAACGTCTTGCTGCAACAGACCAGCAACTACCTTCTCAGCTTGCTTCATGCTAAA
TGTCGTGATGGTGTGCGGATTGAATCTCCTCTTCACACTGTGGATTTCCTGGATGATTTCCACAAGAT
CCTGGAAACCAAGTTTACAGCAAATACATCAGAGCAGTGTATGGACTCAGCAGTGCCACTGGCAGCC
TATAAGTGGCTGGTGTGTTTCTTGCTGGAGGAGAGCCAGAAGAGACTGGAACAGCAGAGGGCCTCA
GGAAACAATGAGTTTGAAGCTCGTAACAACAGCCAGGTATACTACTGTCGTTCCCTGGCCATGGCCT
ATATTGAATACAACTGTCTTCAGAGGTTTCATGAGATGACTACTGATCAGGACACACCAGCTGGTCT
CAGACCTGTACTACGCAAGCTGTGTACCCTTTATGGGCTGTGGAGCCTCAGCAGCCACATGGCCACA
CTCTACCAGGGTAATTACATGAGTGGAGGAAAGCCTGCAGAGTTGGTCCAGATGACAATTGTCACTC
TATGCTCCCAGCTGAAGGATGATGCAGTTGCGTTAGTGGATGTTTTCGCACCTCCTGATTTCATTCTC
AACTCCCCTATTGGATGTGCTGATGGACAGCTTTACAAGAATCTGTGGTCAGCAGTGTTGCAGGGCG
GCCAGGTACTGGAGCGGCCATCCTGGTGGAAAGAGTTCTGCTCAGACAAGCCTGTGGTTGGATCTCT
CCGTCCAAAACTCTAAAACCTACAAGTTAAATTTGTGTGCCCTCAAAATTTCTAACTATACAATTATA
TGTGCTTATTATGGTTAATGGGGTCTTTAAAACCATTCCAACTGAAAATTTAATATGCTACAGCATAC
TAAACTAGTTTGTGAATTCAGGTATAGGTGTAGTCAGGTATCGTTTTAAGTTCCACGGCTCTTTTTTCC
CTATATCTCTGTGTAAAGAGAAATCATGTTCTTTTTTCTAATTATTCTAATGATTTAATTTTAAACTGT
AAAGAAGTAATGCCCTTTTGACAAACAGCAACATAGCATGAAAAAACTTCAGTACAACTCACAGTA
ACACTTTTCTCTTAAATGATCTGCTGATCAAATATCCATGTGGGACTGCTAACAGTTTGCATCTGTAG
ATGTAAATGTCAACATTGTTCCATTTGGAGGCAAATAATGGGCTTCTCCTACAACATCTTTGCTGCTA
AAATCTTGTCTGGCATTAAATTGTAAATTTATTCTAATGTACTCAGCTACTAGGGCACAGAAATTAAA
TTACTGACGATCACTACAACCTGTTCTATAACCTGTAGACTGATTTTGTGAATAACAGTAAACTATCT
CCGTTCTTAACACAGAAACTTCTTTCAAAATGAGGAAATCAATTGTATAATGTATACATGCAATAGC
ATTCTGAGTGCTCAATGTATGAGTATGGCCTCAATATATATATTCTCACTCTTTGACTCCAAAGAAAT

GAATCATATGGGATGTAATATTTTAATGAGTGTAAATTGTGTTATACTGTCTGATGACTTTAAATAAT
GATCACAGTTTTTTAATGATGTAAATTTCTAATCATGTCTTGATAGAATCAAATTAAAGAAATACATT
TACTAATACAACcc

HSL

gacccagcattCTGCGGGACAGACACGGAGCAGAACATCGAGGACGAGGAAGAGGATGCGACTCGCCTCA
GACATGGATTACAAGGTTGTGTTTGCAGCCTTGGAGACAGTATGTGAGGACATCATTTCTGTTATGG
GTGGGCCTTCTGATTTGCCGTACGGCGCTGTTGCCCAGCGCCTGGTCACGTGTATGAAACAGATTCAG
GAGCACGGACAAGCCCTGGAGCCTGTGGTCCGGTGCTTCACCGCCATCTTCCATCATTATGACTTTGA
TGCACAAACGCCTGGAAATGGCTACCGCACCCTGGTCAAGGTCTTGCAGTCTTGCCTTTTGCACATCA
TTCACAAGGGCCACTACATTACCTCAAATTACAACAGTGCCTTCTTCAGGGCGGACCACAATGCCTCT
GAGATGGAGGCGTACTGCAGCGCCCTGTGCCAGCTTCGTGCCCTGATGCACCTCGCTCAGCGGCTGA
TCAACGACAACGACTATGCCCAGCTGTACTCCCTGCAGGAAGGCGACCTCAGCCGTCAGTTCGTGCA
GGAGTACAGCTCCATGCACAAAGCCTGTTTCTATGGCCGCTGTCTGGGCTTTCAGTACTCGCCAGCTC
TACGCCCGTTTCTCCAAACTGTTGTCATAAGCATGGTTTCCTATGGAGAGACGTATGGTAAACATCAG
TCCAGCCTGGGTATGGCAGCCCTCTCCCTGCTCACATCAGGGAAGTACGTCATCGATCCAGAGTTAC
GTGGTGCCGAGTTTGAACGCATTACCCAGAACCTGGACATGCAGTTCTGGAAGTCCTTCTGGAACCT
GACAGAGTCCGGCCTTTTAACAGGCTTCAGCAGGATAGCCTCCAGCCCGGTGCAGGTGAACATCACC
CTGACTTTGCCTCCTGTCACTCTGCGCCTCCCACTCGCCTCAGACCCCAGTCTAACAGCCACCGTGTC
ACCTCCAATAGCCCACTGGGGCCCCGGGCCTGTTCATATGCGTCTGATCTCCTATGAGCTTCGGGAAG
GACAGGAAAGTGAAGAGCTGCTCGCTTTTTCCCGAACCGATCCTCCCCCCATCACAGCATCCCAACT
CCCCTGGGTACAGAAGCAGCCTCGCTCCCCCTGGCTGCTTATCCACTTCCATGGAGGGGGCTTTGTGG
CCCAGACCTCCCGATCCCATGAGAATTATCTTCGGACTTGGTCGAAGGAGCTTAATGTACCTATCCTC
TCCGTGGACTACTCTCTGTCACCCGAAGCTCCTTTTCCCAGAGCTCTGGAGGAATGTTTCTACGCCTA
CTGCTGGGCTCTGAACAACTGTCACCTGCTGGGCTCCACAGCAGAACGGGTTTGTCTGGCAGGTGAC
AGCGCCGGAGGAAACCTCTGCATCACTGTGTCCATGAAGGCCATATCCAACGGCATCCGAGTCCCCG
ACGGCATCATGGCTGCCTACCCCGCCACCTTGCTCACCACCGACGCCTCGCCCTCCCGCCTTCTCACG
CTCATTGACCCGCTGTTGCCTCTAGGTGTTCTCACAAAGTGCCTCAATGCCTATGCAGGTGCAGACTG
TCAGTCGGTGCAGCCTGCAGTGGGAAGCAGCAGTCTGAGCTCTCTGGGTCGAGACACCGCCGTGCTG
CTCGGTGATCTCACCCAGGGAGCCTCCAACTGGATCCAGTCCTTTCTGGACCCCTCGCGAAATACTTC
CGGTGGTGCCCGCTCACTGTCTTTAAGCCTGAGGAGGACCCAGAGCCATGAAATTCGGAGGCCGTCC
ACTCACACCTCCACAGCAAACTGCACCGATCACTTGTATTACCCAGCGGGCTTTGAGCCCCTGCGTTC
AGAGTGCCTGGCTTTTATTCGCCCTACATCCTGTCCTATTATCAGGAACCCGTTTGTGTCGCCCCTGCT
GGCTCCAAACAACCTGCTGAGAGGCCTGCCACCTGTACATATAGTGGCCTCTGCTTTGGATGCCTTGC
TCGATGACTCTGTGATGTTTGCCAAGAAGCTGAAAGACATGGACCAGCCCGTGAGTCTGACGGTGGT
GGAAGACCTGCCTCACGGCTTCCTCAGCCTGTCGCAACTCGCCAAGGAGACGGAGGTCGCTTCAGAA
ATCTGCGTGGAGCAAATGAGGAAGATTTTTCAGCAGCAAAGCCCAACACCTGCCCTCCGCAAACGGC
CAAAGGTGGAAGAGACTTTTCAGAGCACCAATCCGTCTGGCTGATTTATTAACCGTCGTTCTTGGGC
ACTTTGTTGTTGTTGGCTGACGTAATGTATTGAGAGAGATGCAATGAATGTAAGAGAGGGAAACAAG
TGAAGGATAATTATGAGGAAGCACAGGGAAGCTGCACTTTAAACCTTTTCAGCACAACACTTTCTGT
AGGCAACAACGATCGAGAGATCTGTAACAGCTCAAAAATGATCCTAAACGATGATAAAAAAGGAAA
CAAGTATTTTTCTACCAAGATTTATGTGTTCAAGATCCAGATTAAATGCACAATGAGAATATAACATA
TGTAACAAAAACTTAACCGTAACCAGCCCTCACAGTAATTTCTTAACTGATAAACGTCAGTAATGGA
AGCTTCTGTGCCTTGAATTTAAAGTGCTGCTGTAAAAAAAAAAAAAAGACTAAAAAAAAAAAAAAA
AAAACCTACATCACCAAACTGACCAGGATTAAAGGTTTACTTGTACAGGAAACACAGTCCAGATGCA
GTGTATTTCTATTATTAAGATTATATTTGGATTAAATGTGGAATCTGGTTTTGCCAGTTTGCCACATGT
TTACAATATATTGTTAAGGAGGCCTTCACATTGTGTTTACCAGCAGTATGTGGATTATGGCAGTGGAC
GTGAAAGAAACTTGTAATAATGTAAATTAATTCTCAGTATTTCTTAATAATAGTATTTATCACTGTGT

TGTTATATGTAAAATCTGAATTAAATCACAACTtc

UCP1

gcCCTTTCTGTGGAGCCGGTCTTTTATGGATTATAATCCGGCCAATCAAATCCTGCCCATCCGGTAACA
AAAAACTGAGGATCAACAGGAGCCTTTAAATTTTGGACACACAATTTTTAGGAAACATGGTGGGACT
TAAACCTTCAGATGTTCCCCCTCCACTTGGGGTGAAGATGGCGAGTGCTGGGGCAGCAGCCTGTATA
GCTGACATTGTAACATTTCCTCTGGACACAGCCAAAGTCAGACTACAGATTCAGGGAGAGAAGACGG
CAGTGGGAGGCATCCGCTACAGAGGCGTGTTTGGGACGATCACCACCATGATCAGAACAGAAGGGC
CCAAGTCTCTGTACAATGGGCTGGTAGCCGGGCTGCAGAGACAATTGTGCTTTGCCTCCATAAGGAT
TGGCCTCTATGACAATGTCAAAAACTTCTACACTGGTGGCAAGGACAATCCTAATGTGTTCATTCGTA
TCCTGGCTGGCTGCACTACGGGTGCAATGGCAGTGTCCTTTGCACAGCCCACAGATGTGGTCAAGGT
TCGATTCCAAGCCCAGATGAACCTGGACGGTGTGGCCCGTCGCTACACCGGCACCATGCAGGCCTAC
AGACACATCTTCCAGAATGAGGGCCTTCGTGGACTCTGGAAAGGGACGTTACCCAACATCACAAGAA
ACGCCTTGGTCAACTGCACAGAGCTGGTTACGTATGATCTGATCAAGGAGGCCGTCCTTAGACACAA
CCTGTTGTCAGACAATCTGCCTTGCCACTTTGTATCTGCATTTGGTGCTGGTTTTGTTACCACGGTGAT
TGCTTCCCCAGTAGATGTGGTGAAAACTAGATACATGAACTCACCTCCAGGCCAGTACAAGAGCGCT
ATCAACTGCGCCTGGACCATGATGACCAAAGAGGGTCCGACGGCTTTCTACAAAGGATTTGTGCCCT
CGTTCCTGAGGTTGGGATCGTGGAACGTTGTGATGTTTGTCTCATTCGAACAAATCAAGAGAGCCAT
GATTGTCACAAAGAAGAAGATCGAGGCCCCAAACTGAGCTTTAGACTGAAAAACTTGAACCACCAC
TGCTGGGTTGGAACATGAAGAAAGCCTAATGAATATCATATCACGCTTTAGTTTTATTCTTTTTTTTGT
CTTGTGGTGAAAAAGAGGAAATTCCTTTGTTGAAAAAGGAATACTGGACATCTTGTTTGTTGACTTTA
TATTGAACCAATGTATGAAAGAAATGCAGATGTACCTTTATATTTATAATTTTTCATAATGAACCCAT
TTCAAATTTAATGGGAATGAATGGTGTGAACAGGAAGTTGTGATAAACTCCTTTGGGAAGTTGAGTT
TTCACTTTAAGACCTATAAATCTAAATCTGCCAATTATACATTTAAGGCAGCTTAAACCATTGCACCT
TTATACTGGAAATAATACTGGTGCCCATAATAAAATGTAAAACAGGTTTATGTAAACCTGAAATATA
TTTGACTTGTTTTACAGACCCTGTGCTTGCTTATTAACTTCCACCACACGAAACACATCTCAAAACAT
CAAAATGTGTATTTACTAAATCTGAGTAACATTTGTTAATCATTATACACCTTCCTCAAAAGCAAAAG
CATAAAAATAAGGTAATAAAAATGCTGTTATCAGTCTTCAGACATACAAATTTATGGGGGCAAACAA
CCATTTTGAGATCCACATTACCTATATTACAGAATACAGATGACATCAATGGGGATACGTGCACATA
CATTAAAGTTTCAGCCATTCTCTGAACGTAAAAATTAAATTCCTGAGCAGGACAACCGTGAAAACAC
ACAAGTCTTGTTCACATAAATACAAACAAGGCAGCTACAAATATGGTGATTAATCGCCATTTCTCAA
TAAGTGTCTTTTAACTGCAACAATTTGGCTTCTTGCTCACTGAAAGTGCTTGAACCCTTCGCTACAGA
TCCCTGCAGCAGCTCTCGATTCCCCTTTACGAAGCTTGATTAAACACCGGAATGATCATTTATTTTTG
AGGTATAGGGTGTTAGATTTGTGCTCTATTTAAAAACCTTTAACTATCACTGTGAAGAAATAAACAG
AGAATGACTAACAAATCACAAAACCAAATATAAGAGGATTTATTTGTTTGAATGTTTGAGGAAAATG
ACCGACCAGCTTCAGTCCGCTTTTACGCTTCTCGCCTTCTTCTGCCTGAAATGATGTCAGAGGCTGCA
GAGGATCAGCAACTATCTCTGAATAATGTTAGTTTTTAGAGCAGACAGTGAGTGTGAGGGATACATC
GGGGTCCTGTAGATGCATCTTGACGATGTCATGGAATTTCTCTCGGTACTGATTGAACTGCATGATGC
TTTCCGGCTCCTCTGCCACCCAGAATTTATCAAATTCGTATGCCAGATAACCTGGAAGACAAATAAAT
AATTCTCTATAAAAACTGC

MIFgACACTACATAGAGGAAGAGACGTCTTTAGTCTTGTCTTCCGCCTGTGAGCAGAGTAGTGGTAGC
ATCTGCGGTTTGTGAAAGGAGAGAAGGACTACATTGCGATCATGCCGATGTTTGTGGTGAACACCAA
CGTGGCCAAATGCGATGTGCCGGCGGGTCTGCTGTCCGAGGCTACTGAGGAGCTGGCCAAAGCCATG
GGCAAACCTGCGCAGTACATTGCTGTGCATATCAACCCTGACCAAATGATGATGTTTGGAGGAAAAG
GAGACCCCTGCGCCCTCTGCTCCCTCCACAGTATTGGCAAGATCAGTGGTGCACACAACAAGCAATA
CTCTAAACTCCTATGTGGACTGCTCAACAAACACCTGGGCATCTCTGCTGACAGGATTTATATTAACT
TTGTAGACATGGATGCAGCCAACGTGGCCTGGAACAACACTACTTTTGGCTGAGATGGAGGTCAACA
CACTGAAATAGAAGATCATTCCAGTATTTTCCTCCATGTATGAACAGAATTAAAAGAGACAAAATGA
AAAAGGAAAATACCTGGTCATTCACTGGTCAATATATTTTATGGCTATATTACAAGCACTTAACTGG
ACCATTTAGCTTTGCACTGATCCTTCTCAATTCGAATGCAATAAAGTAAATCCTGTATAACATCAG

B2M

GGAGTTCATTTTCGCTCTTTACAGGACAAGCAGCACGCTTCTCATCTCCTTGCCAAACTACCAAAGAA
CATGAAGTTTGTTCTGTGCCTTGCAGCTCTGGTAGCTGTCTGCTACTCAGAGGACTCCAAGCACACTC
CACCCAAGGTTCAGGTGTACAGCCGTGACCCTGGAAAGTTCGGCAGTAGTAACGTCCTGATTTGTCA
TGTGAGTGGCTTCCACCCACCTGACATCACCATCCAGCTCATGAAGGATGGAGTGGAACTTCCTAAT
GCCAAGCAGACTGACCTGGCCTTCAAACAGGACTGGCACTTCCATCTGACCAAGAACGTGGACTTCA
CCCCCCTGGATGGACAGAAGTACATGTGCAGGGTCACTCATGGGCAGACAGTCAGAGACTATGCCTG
GGAGCCAAACATGTAACTCATCTGTCCTCAGCTACAGCAGTATCTGTCATTAACATCTCCAATCAACA
GGATTCATGTTATTCGAACCTTCTCACCTTTCTTTTCACACACATAAACATCAGCACCATATGAAGAG
GAATTACAATCATCAATATATTCTGATAATTCATGGGGTCCAGGTCATTTAGTTGGCTATCTGTTTAG
ATTGCTAAAATGTTTTAGATTACAACTTATTTTCTGCTCTTTGATACTGATAAGTGTATATATTAAACT
AAAGTGGTGAGAAATGCCTTTGCTCTTTCACATGTTTTTCTTTTTTAAAGGCTTTAACTGTTTCTTCTA
CCTAAATGAGGCAATCAGATGATAAAAAAAAAAAGAATGGGGGTTTCTTTCTGGGCTATAAATGTGT
GTTGTATAATTTGGATTTGCTATTTAAAGAATTTGTTCATGCCCTGTATGAAATAAATGCCTCTTGAA
AATTC
