# Supplementary material for: PPAR Signaling Maintains Metabolic Homeostasis under Hypothermia in Freshwater Drum (Aplodinotus grunniens)
Source: Metabolites. 2023 Jan 8;13(1):102. doi: 10.3390/metabo13010102 (PMC9865675; doi:10.3390/metabo13010102)
Supplement: Supplementary file 1 [file metabolites-13-00102-s001.zip › Figure S1 GO and KEGG enrichments of DEGs in Freshwater drums liver induced by 2 days cold stress.pdf]

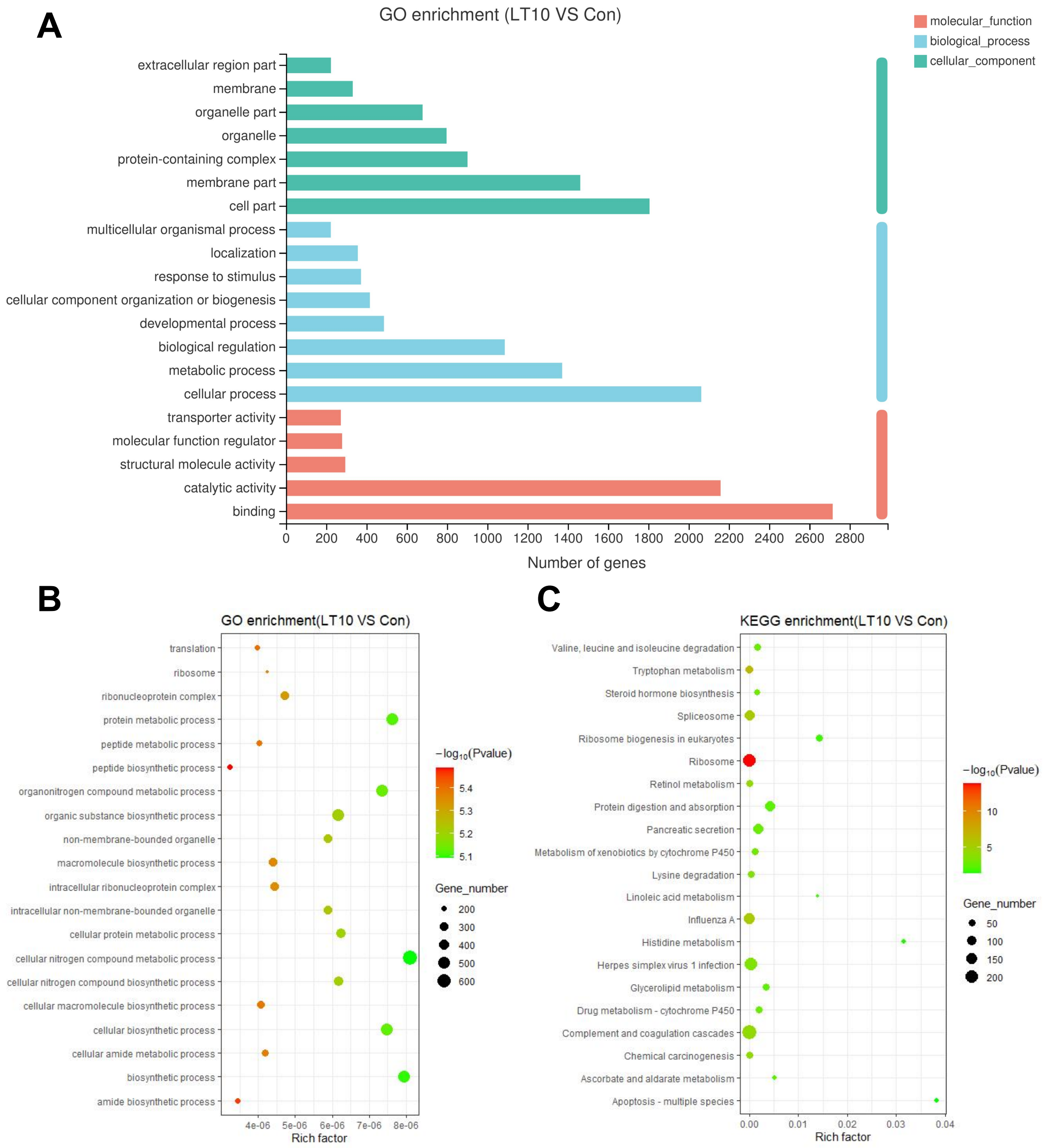

Figure. S1**. GO and KEGG enrichments of DEGs in Freshwater drums liver induced by 2 days cold stress.**

A), GO catalogs of DEGs; B), GO enrichment of DEGs; C), KEGG enrichment of DEGs.
